# Effects of Linagliptin on Liver Enzymes in Patients with Type 2 Diabetes Mellitus: A Systematic Review and Meta-Analysis

**DOI:** 10.3390/life16081343

**Published:** 2026-08-16

**Authors:** Moragot Chatatikun, Ratana Netphakdee, Aman Tedasen, Jason C. Huang, Wiyada Kwanhian Klangbud, Jongkonnee Thanasai, Atthaphong Phongphithakchai

**Affiliations:** 1 Department of Medical Technology, School of Allied Health Sciences, Walailak University, Nakhon Si Thammarat 80160, Thailand; moragot.ch@wu.ac.th (M.C.); aman.te@wu.ac.th (A.T.); 2 Biomedical Sciences and Health Innovations (International Program), School of Allied Health Sciences, Walailak University, Nakhon Si Thammarat 80160, Thailand; ratana.ne@mail.wu.ac.th; 3 Research Excellence Center for Innovation and Health Products (RECIHP), Walailak University, Nakhon Si Thammarat 80160, Thailand; 4 Department of Biotechnology and Laboratory Science in Medicine, National Yang Ming Chiao Tung University, Taipei 112304, Taiwan; jasonhuang@nycu.edu.tw; 5 Medical Technology Program, Faculty of Science, Nakhon Phanom University, Nakhon Phanom 48000, Thailand; wiydakhanhian@gmail.com; 6 Faculty of Medicine, Mahasarakham University, Mahasarakham 44000, Thailand; jongkonnee@msu.ac.th; 7 Nephrology Unit, Division of Internal Medicine, Faculty of Medicine, Prince of Songkla University, Songkhla 90110, Thailand

**Keywords:** linagliptin, dipeptidyl peptidase-4 inhibitors, type 2 diabetes mellitus, liver enzymes, alanine aminotransferase, aspartate aminotransferase

## Abstract

Type 2 diabetes mellitus (T2DM) is often linked to metabolic dysfunction-associated steatotic liver disease (MASLD), a metabolic liver condition defined by excessive lipid deposition within hepatocytes and is frequently accompanied by increased circulating liver enzyme levels. Dipeptidyl peptidase-4 (DPP-4) inhibitors, such as linagliptin, may exert pleiotropic effects on hepatic metabolism; however, their effects on liver enzyme profiles remain uncertain. This systematic review and meta-analysis aimed to evaluate the impact of linagliptin on liver enzymes, including aspartate aminotransferase (AST), alanine aminotransferase (ALT), alkaline phosphatase (ALP), and gamma-glutamyl transferase (GGT), in patients with T2DM. A systematic literature search was performed across five electronic databases up to 11 May 2026 following the PRISMA 2020 guidelines. Randomized controlled trials and cohort studies were included, and pooled mean differences (MDs) were calculated using random-effects models. Eight studies involving 1262 participants were analyzed. Linagliptin was associated with a modest reduction in AST (MD −1.58 U/L, 95% CI −2.85 to −0.31) with low heterogeneity, whereas the change in ALT was not statistically significant (MD −1.86 U/L, 95% CI −4.14 to 0.42), and substantial heterogeneity was observed. No significant effects were observed for GGT, while evidence for ALP was limited to a single study and was insufficient to determine the effect of linagliptin. Overall, linagliptin demonstrated limited and inconsistent effects on liver enzyme profiles.

## 1. Introduction

Type 2 diabetes mellitus (T2DM) continues to represent a major and growing global health burden, with increasing emphasis on identifying metabolic complications beyond traditional vascular outcomes. The liver has emerged as a key organ involved in the pathophysiology of diabetes, serving both as a metabolic regulator and a target of disease-related injury [1]. While macrovascular and microvascular pathologies have historically dominated clinical focus, contemporary evidence underscores the liver as both a primary target organ and a metabolic driver of diabetes-associated complications [2,3]. Chronic hyperglycemia and insulin resistance promote enhanced lipolysis and free fatty acid flux to the liver, leading to intrahepatic lipid accumulation. This condition is currently classified as metabolic dysfunction-associated steatotic liver disease (MASLD) or non-alcoholic fatty liver disease (NAFLD), which is highly prevalent among individuals with T2DM [4,5,6,7]. Over time, persistent lipotoxicity triggers inflammatory cascades, mitochondrial distress, and hepatocyte ballooning [8]. Progressive lipid accumulation contributes to hepatocellular stress, inflammatory activation, and structural injury, ultimately resulting in increased circulating liver enzymes such as aspartate aminotransferase (AST) and alanine aminotransferase (ALT) [9]. Consequently, elevated circulating transaminases serve as vital, accessible clinical biomarkers for monitoring hepatic necroinflammation and metabolic liver injury [10].

Dipeptidyl peptidase-4 (DPP-4) inhibitors, including linagliptin, improve glucose homeostasis by preventing the breakdown of incretin hormones, such as endogenous glucagon-like peptide-1 (GLP-1) and glucose-dependent insulinotropic polypeptide (GIP), thereby stimulating insulin secretion and reducing glucagon activity [11]. In addition to improving glycemic control, growing evidence suggests that these agents may influence hepatic metabolism through modulation of lipid handling, inflammatory signaling, and oxidative stress pathways. Linagliptin exhibits a unique pharmacokinetic profile, characterized by high affinity for DPP-4 and predominantly non-renal elimination, which may offer advantages in specific patient populations. Despite mechanistic evidence supporting potential hepatic benefits, clinical findings regarding the effects of linagliptin on liver enzyme levels remain inconsistent across studies [12,13,14]. Mechanistically, experimental and clinical studies suggest that linagliptin may reduce hepatic fat accumulation through inhibition of lipogenic pathways and promotion of fatty acid β-oxidation [15,16]. In parallel, inhibition of DPP-4 activity has been linked to suppression of inflammatory signaling pathways and decreased macrophage infiltration within hepatic tissue, including nuclear factor-kappa B (NF-κB), and reduced macrophage infiltration within hepatic tissue, thereby limiting inflammatory injury and oxidative stress [17,18]. However, despite these mechanistic insights, the magnitude and consistency of linagliptin’s effects on liver enzyme levels remain variable across different clinical populations and study designs, highlighting the need for further quantitative synthesis and evaluation.

Despite growing evidence suggesting reported outcomes vary across studies, likely reflecting heterogeneity in study design, population characteristics, and outcome measurement. In addition, most available studies have focused primarily on glycemic control, with limited emphasis on liver-specific biomarkers, including aspartate aminotransferase (AST), alanine aminotransferase (ALT), alkaline phosphatase (ALP), and gamma-glutamyl transferase (GGT), thereby limiting the understanding of its overall hepatic effects. Furthermore, small sample sizes, short follow-up durations, and heterogeneous comparator therapies further hinder the interpretation of existing evidence. Therefore, a comprehensive synthesis is needed to clarify these discrepancies. The present systematic review and meta-analysis evaluated the effects of linagliptin on liver enzyme levels, including AST, ALT, ALP, and GGT, in patients with type 2 diabetes mellitus, and assessed the consistency and certainty of these findings across variations in study design, patient populations, and treatment duration.

## 2. Materials and Methods

### 2.1. Study Protocol and Selection Criteria

The review followed the Preferred Reporting Items for Systematic Reviews and Meta-Analyses (PRISMA 2020) guidelines [19] and its protocol was prospectively registered with PROSPERO (CRD420261384229) on 1 May 2026 to ensure methodological transparency. The eligibility criteria were predefined according to the PICOS framework, encompassing population, intervention, comparison, outcome, and study design, as detailed in Table 1.

### 2.2. Search Strategy

Relevant studies were identified through searches of five electronic databases: PubMed, Web of Science, Embase via Ovid, the Cochrane Library, and Scopus from inception to 11 May 2026. Search terms combined controlled vocabulary (MeSH terms) and relevant keywords related to linagliptin and liver enzyme outcomes. The detailed search strategies for the databases are provided in Supplementary Table S1–S5. Studies were eligible regardless of publication year, but the review was limited to articles published in English.

### 2.3. Eligibility Criteria

Studies were considered eligible when they fulfilled the following criteria: (1) adults aged ≥ 18 years; (2) patients diagnosed with T2DM; (3) those with or without coexisting liver conditions (e.g., non-alcoholic fatty liver disease (NAFLD)); (4) treatment with linagliptin; (5) presence of a control group receiving placebo, standard care, or other antidiabetic agents (e.g., metformin, insulin, or other DPP-4 inhibitors); (6) study designs including RCTs, non-randomized controlled trials, and cohort studies; (7) reporting of liver enzyme outcomes, including AST, ALT, ALP, or GGT; and (8) full-text articles published in English.

The exclusion criteria were as follows: (1) participants aged < 18 years; (2) patients with type 1 diabetes mellitus (T1DM) or gestational diabetes mellitus (GDM); (3) studies not employing eligible designs, including RCTs, non-randomized controlled trials, or cohort studies; (4) absence of an appropriate control group; (5) studies not reporting relevant liver enzyme outcomes (AST, ALT, ALP, or GGT); (6) studies published in languages other than English; and (7) publications without sufficient extractable data, including case series, commentaries, case reports, editorials, letters, and conference abstracts.

### 2.4. Study Selection Process

All records identified through the database searches were transferred to EndNote for reference management and duplicate removal (version 20; Clarivate, Philadelphia, PA, USA), followed by additional manual verification. Study selection was performed in two stages. Initially, two reviewers (M.C. and R.N.) independently assessed titles and abstracts according to the predefined eligibility criteria [20]. Agreement between reviewers was quantified using Cohen’s kappa (κ) statistic with corresponding 95% confidence intervals (CIs). The strength of agreement was interpreted as follows: κ = 0.41–0.60 indicated moderate agreement, 0.61–0.80 substantial agreement, and 0.81–1.00 almost perfect agreement [21]. Disagreements were discussed between the reviewers until consensus was reached; unresolved issues were referred to a third reviewer (A.P.).

### 2.5. Data Extraction and Management

Two reviewers (M.C. and R.N.) independently extracted the data from the included studies using a standardized data extraction form. The following study characteristics were extracted from each eligible study: first author, publication year, country and study design; population characteristics (sample size per group and the presence of underlying NAFLD/MASLD); intervention details (linagliptin dosage and treatment duration) and control conditions (placebo or active comparators with corresponding doses); and hepatic outcomes, including post-treatment levels of AST, ALT, ALP, and GGT, reported in U/L as mean ± standard deviation (SD) for both intervention and control groups. The extracted data were independently verified, with discrepancies resolved through discussion or, when necessary, consultation with a third reviewer (A.P.).

### 2.6. Quality, Risk of Bias, and Certainty of Evidence Assessment

Two reviewers (M.C. and R.N.) independently assessed the methodological quality and risk of bias of the included studies. Randomized controlled trials (RCTs) were assessed using the Cochrane Risk of Bias 2 (RoB 2) tool, which examines five domains: the randomization process (D1), deviations from intended interventions (D2), missing outcome data (D3), outcome measurement (D4), and selection of reported results (D5) [22].

The Newcastle–Ottawa Scale (NOS) was used to assess the quality of non-randomized and observational cohort studies, which considers three key domains: participant selection, comparability of study groups, and outcome assessment [23]. Observational studies with NOS scores ≥ 7 were classified as high quality. To facilitate consistent interpretation, NOS ratings were further categorized according to the Agency for Healthcare Research and Quality (AHRQ) criteria into good, fair, or poor quality, based on predefined thresholds across selection, comparability, and outcome domains [24]. Reviewer disagreements were discussed to reach consensus, with unresolved issues referred to a third reviewer when necessary (A.P.).

The certainty of evidence for each pooled outcome (AST, ALT, GGT, and ALP) was assessed using the Grading of Recommendations Assessment, Development and Evaluation (GRADE) framework [25]. Evidence quality was classified as high, moderate, low, or very low based on evaluation across five domains: risk of bias, inconsistency, indirectness, imprecision, and publication bias.

### 2.7. Data Synthesis and Statistical Analysis

A quantitative synthesis was performed when comparable outcomes were reported by at least two studies. Continuous variables were analyzed using mean differences (MDs) with corresponding 95% confidence intervals (CIs). When necessary, summary statistics reported as medians and interquartile ranges, approximate means and standard deviations were calculated using the method proposed by Luo et al. [26].

Between-study variability was evaluated using Cochran’s Q test and quantified with the *I*^2^ statistic. A random-effects model (DerSimonian–Laird method) was applied to account for potential heterogeneity [27]. The *I*^2^ values were interpreted according to established thresholds: 0–25% indicated low heterogeneity, 26–50% moderate heterogeneity, 51–75% substantial heterogeneity, and >75% considerable heterogeneity [28]. Subgroup analyses were performed to investigate potential sources of heterogeneity according to the following characteristics: (1) study design, (2) patient population (T2DM vs. NAFLD with T2DM), and (3) treatment duration. The robustness of the pooled estimates was assessed by sensitivity analysis using a leave-one-out approach, whereby each study was sequentially excluded to evaluate its influence on the overall results [29].

Publication bias was assessed qualitatively through visual inspection of funnel plot symmetry and quantitatively using Begg’s rank correlation and Egger’s regression tests, with statistical significance defined at *p* < 0.05 [30,31]. All statistical analyses and graphical outputs, including forest plots, were generated using RStudio 4.6.0 (Posit Software, PBC, Boston, MA, USA). For all pooled analyses, statistical significance was defined as a two-tailed *p*-value below 0.05.

## 3. Results

### 3.1. Search Results

**Figure 1:**
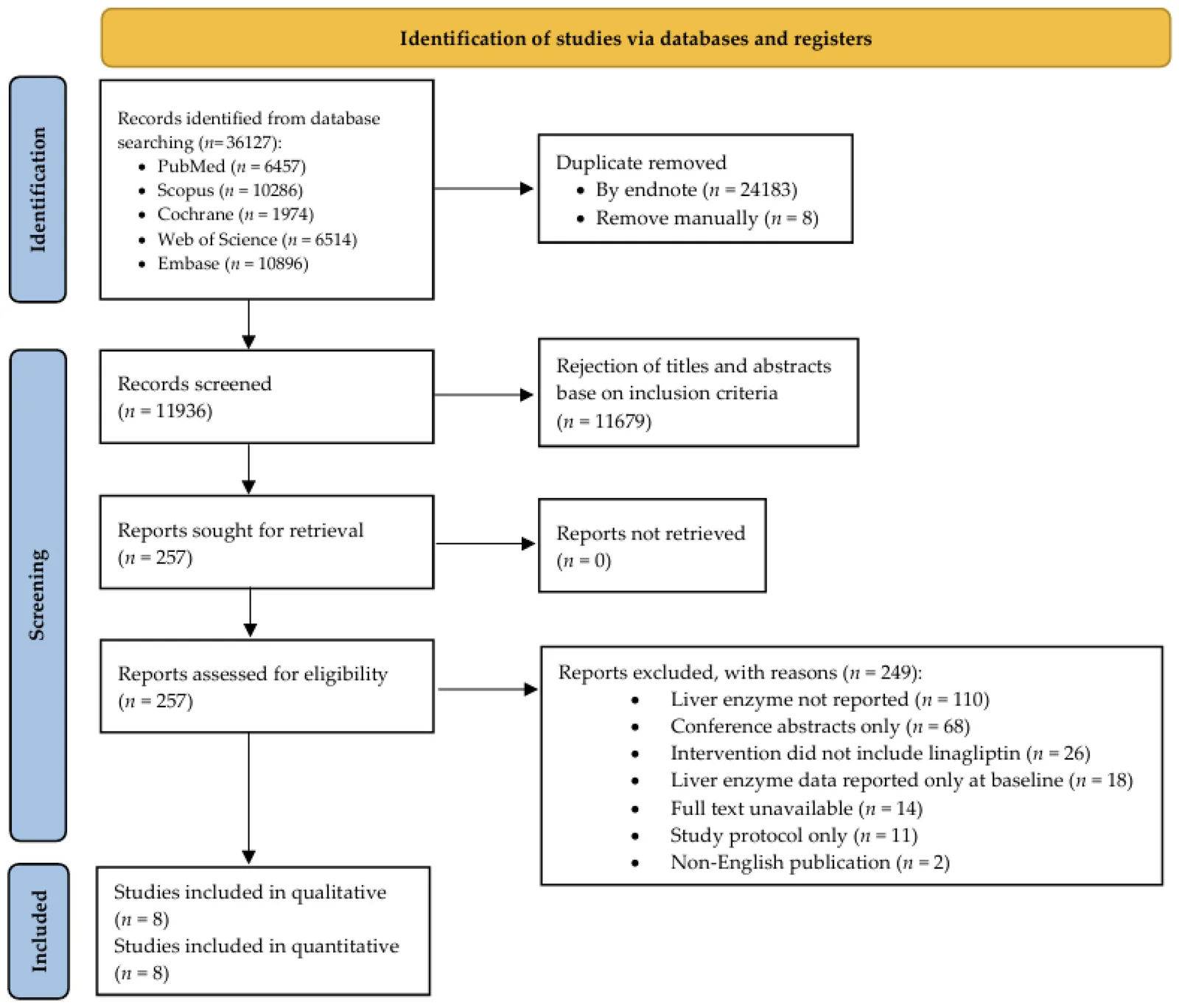
PRISMA flow diagram illustrating the study identification and selection process.

Database searches yielded 36,127 records in total including PubMed (*n* = 6457), Scopus (*n* = 10,286), Cochrane Library (*n* = 1974), Web of Science (*n* = 6514), and Embase (*n* = 10,896). Following duplicate removal using EndNote (*n* = 29,633) and additional manual screening (*n* = 8), a total of 11,936 records remained for title and abstract screening. Of these, 11,679 records were excluded based on the predefined eligibility criteria. Subsequently, 257 reports were sought for retrieval, and all were successfully obtained and assessed for eligibility. Among these, 249 studies were excluded for the following reasons: liver enzyme outcomes not reported (*n* = 110), conference abstracts only (*n* = 68), intervention not including linagliptin (*n* = 26), liver enzyme data reported only at baseline (*n* = 18), full text unavailable (*n* = 14), study protocols only (*n* = 11), and non-English publications (*n* = 2). Ultimately, all eight studies contributed to both the qualitative and quantitative syntheses as shown in Figure 1 [32,33,34,35,36,37,38,39]. The inter-rater reliability between the two independent reviewers was quantified; the title–abstract screening phase demonstrated very good agreement (Cohen’s κ = 0.94), and the full-text assessment phase also showed very good agreement (Cohen’s κ = 0.83), confirming high methodological consistency across the selection process as shown in Supplementary Tables S6 and S7.

### 3.2. Characteristics of the Included Studies

Table 2 summarizes the characteristics of the eight studies included in this study, comprising six RCTs [32,33,35,37,38,39] and two cohort studies [34,36], conducted across Italy (*n* = 1) [32], Japan (*n* = 4) [33,34,35,36], China (*n* = 2) [37,39], and Austria (*n* = 1) [38]. The majority of studies enrolled patients with T2DM (*n* = 7) [32,33,34,35,36,38,39], while one study included patients with concurrent NAFLD and T2DM (*n* = 1) [37]. The duration of linagliptin treatment ranged from 3 to 24 months. Across the included studies, the total number of participants in the linagliptin (intervention) group was 571, while the total number in the control groups was 691. For studies with multiple comparator arms, the shared intervention group was evenly divided across comparisons to prevent the same data from being counted more than once in the meta-analysis. Comparator interventions included placebo (*n* = 3) [32,38,39], metformin (*n* = 1) [35], glimepiride (*n* = 1) [37], voglibose (*n* = 1) [33], and other DPP-4 inhibitors, including anagliptin and sitagliptin (*n* = 2) [34,36]. All included studies reported liver enzyme outcomes, with AST and ALT evaluated in all studies (*n* = 8) [32,33,34,35,36,37,38,39], while alkaline phosphatase and gamma-glutamyl transferase were additionally assessed in a subset of studies (*n* = 2) [33,37]. Outcome data were predominantly reported as mean ± standard deviation (*n* = 7) [33,34,35,36,37,38,39]. One study reporting results as median with interquartile range (*n* = 1) [32], the reported values were converted to the approximate mean and standard deviation using the method described by Luo et al., 2018, as shown in Supplementary Table S8 [26]. The original and converted values for Cioffi et al. (2021) are provided in Supplementary Table S9 [32].

### 3.3. Results of the Quality Assessment

**Figure 2:**
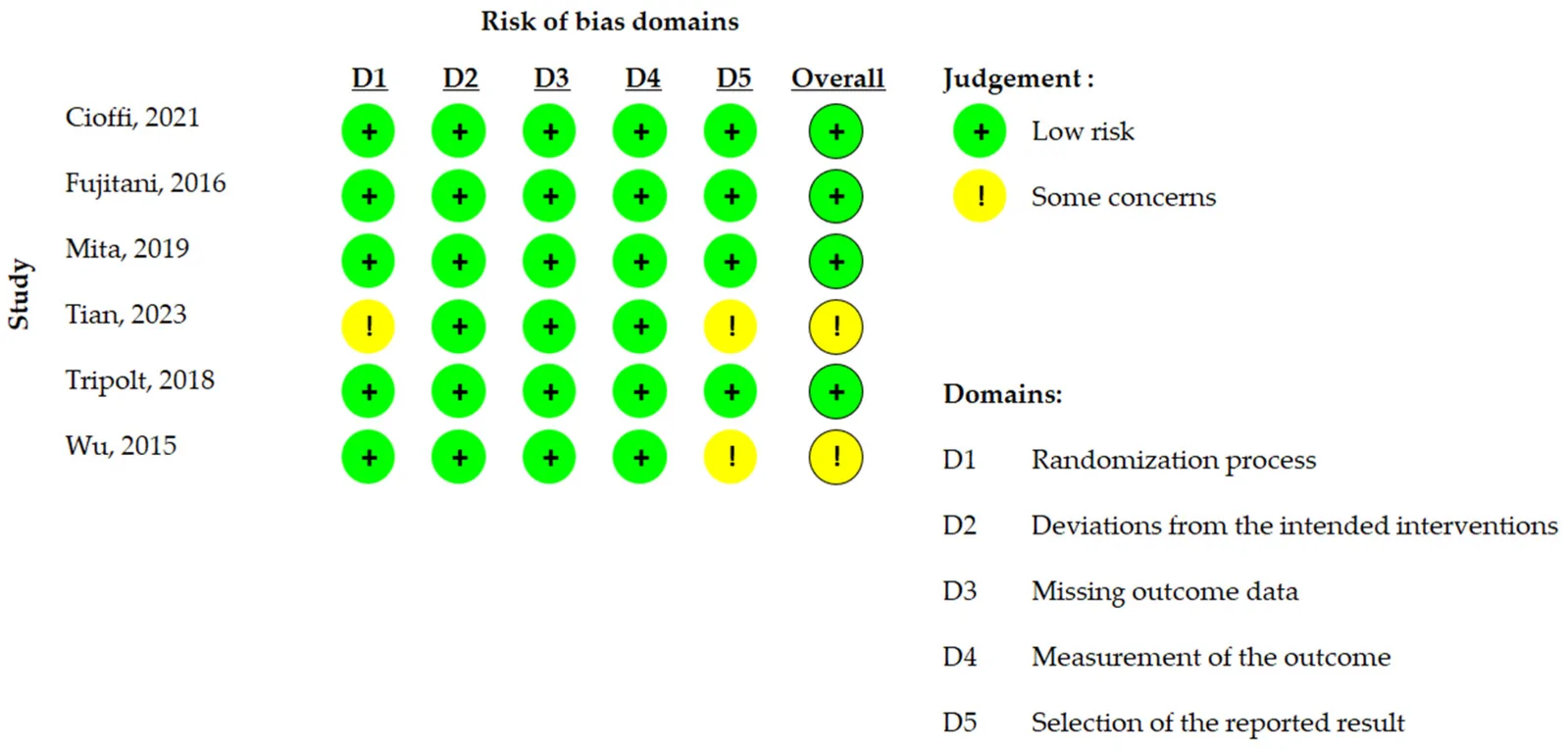
The included randomized controlled trials were assessed for risk of bias using the Cochrane Risk of Bias 2 (RoB 2) tool [32,33,35,37,38,39]. The evaluated domains include the randomization process (D1), deviations from intended interventions (D2), missing outcome data (D3), outcome measurement (D4), and selection of reported results (D5). Risk of bias is classified as low risk (+, green), some concerns (!, yellow), or high risk (−, red).

The risk-of-bias assessment indicated that most included randomized controlled trials (RCTs) had a low risk of bias across all domains, as shown in Figure 2 and Table 3. Cioffi et al., 2021; Fujitani et al., 2016; Mita et al., 2019; and Tripolt et al., 2018, were consistently rated as low risk of bias across all five RoB 2 domains, supporting an overall low risk judgement [32,33,35,38]. In contrast, Tian et al., 2023, and Wu et al., 2015, showed some concerns, primarily due to potential issues in the selection of reported results and missing outcome data, leading to an overall judgement of some concerns rather than high risk [37,39]. Importantly, no study was classified as having a high risk of bias.

For the cohort studies, quality assessment using the Newcastle–Ottawa Scale (NOS) indicated that both studies were of good methodological quality according to Agency for Healthcare Research and Quality (AHRQ) thresholds as shown in Table 3 and Supplementary Table S10. Hamasaki and Hamasaki, 2018, achieved a total score of 7 out of 9 stars, demonstrating strong selection criteria but limited comparability and moderate outcome assessment [34]. Nishida et al., 2020, achieved the maximum score of 9 out of 9 stars, indicating robust performance across selection, comparability, and outcome domains [36]. Overall, the included cohort studies were assessed as having a low risk of bias and good methodological quality.

### 3.4. Primary Outcomes

#### 3.4.1. Effect of Linagliptin on AST

**Figure 3:**
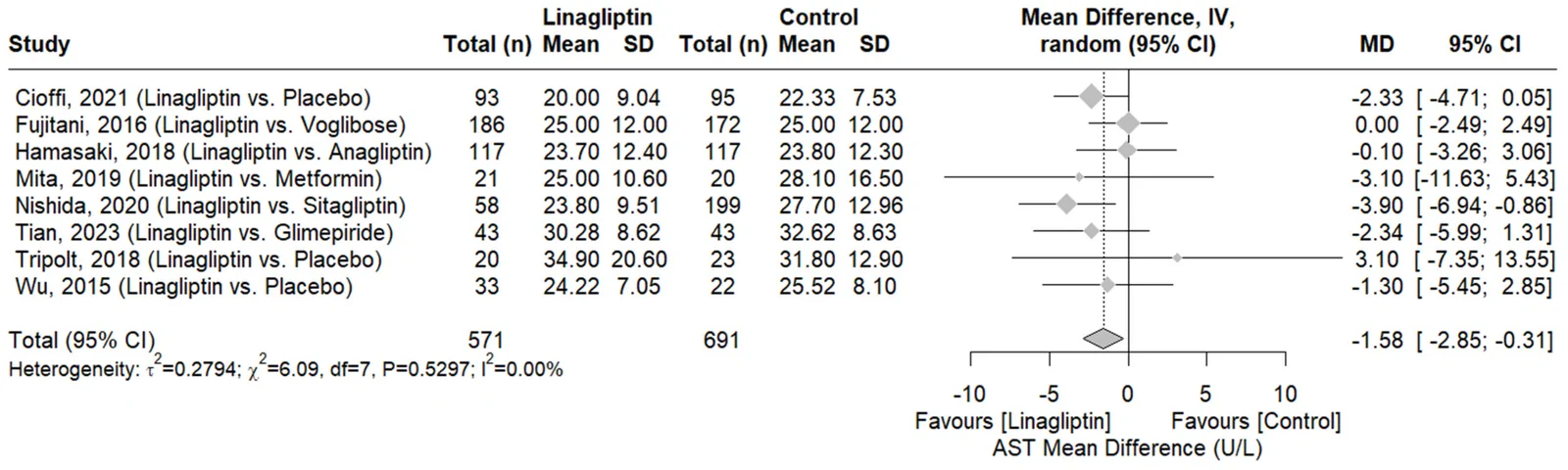
Forest plot of the effect of linagliptin on aspartate aminotransferase (AST) levels compared with control treatments [32,33,34,35,36,37,38,39]. Effect estimates are expressed as mean differences (MDs) with 95% confidence intervals (CIs) using a random-effects model. Individual study results are represented by squares proportional to study weight, with horizontal lines indicating the corresponding 95% CIs. The pooled estimate is illustrated by a diamond, with its width reflecting the 95% CI. The vertical line at zero denotes no difference between groups. Negative MD values favour linagliptin, whereas positive values favour the control group.

The pooled analysis of eight studies indicated that compared with control interventions, linagliptin treatment was associated with a significant reduction in AST levels, as shown in Figure 3 [32,33,34,35,36,37,38,39]. The pooled mean difference (MD) was −1.58 U/L (95% CI: −2.85 to −0.31), indicating that AST levels were lower in the linagliptin group. The included studies showed low levels of heterogeneity (*I*^2^ = 0%, *p* = 0.5297), suggesting comparable findings across studies. Most individual studies showed a trend favouring linagliptin, although several comparisons were not statistically significant, with confidence intervals crossing zero. Overall, these results indicate that linagliptin modestly but consistently improves AST levels compared with placebo or active comparators.

#### 3.4.2. Effect of Linagliptin on ALT

**Figure 4:**
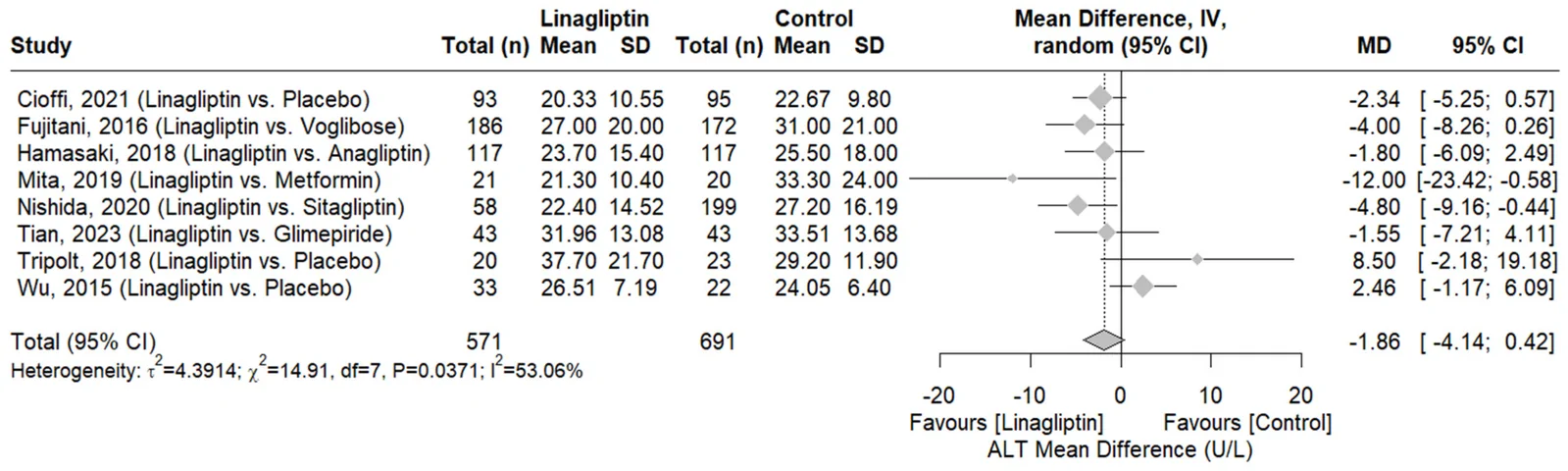
Forest plot illustrating the effect of linagliptin on alanine aminotransferase (ALT) levels compared with control treatments [32,33,34,35,36,37,38,39]. Effect sizes are expressed as mean differences (MDs) with 95% confidence intervals (CIs) based on a random-effects model. Individual study estimates are shown as squares with sizes proportional to their weights, and corresponding 95% CIs are represented by horizontal lines. The pooled estimate is depicted by a diamond, with its width indicating the 95% CI. The vertical reference line at zero denotes no difference between groups. Negative MD values favour linagliptin, whereas positive values favour control.

Pooled analysis of eight studies indicated that linagliptin tended to reduce ALT levels compared with control treatments as shown in Figure 4 [32,33,34,35,36,37,38,39]. The pooled MD of ALT was −1.86 U/L (95% CI: −4.14 to 0.42; *p* > 0.05), representing no statistically significant difference. Substantial heterogeneity was observed across studies (*I*^2^ = 53.06%, *p* = 0.0371), suggesting variability in effect estimates. Although several individual studies demonstrated reductions in ALT favouring linagliptin, the overall pooled estimate crossed the null value, indicating no significant difference between groups.

### 3.5. Secondary Outcomes

#### 3.5.1. Effect of Linagliptin on GGT

**Figure 5:**
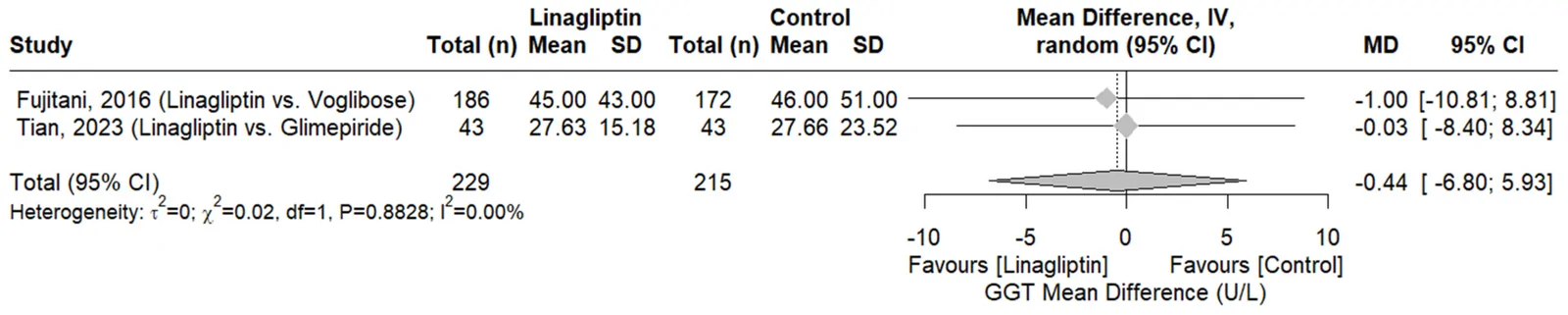
Forest plot of the effect of linagliptin on gamma-glutamyl transferase (GGT) levels compared with control treatments [33,37]. Results are reported as mean differences (MDs) with 95% confidence intervals (CIs) based on a random-effects model. Squares denote individual study estimates (weighted by size), and horizontal lines represent 95% CIs. The diamond indicates the pooled effect, and the vertical line at zero represents no effect. Negative values favour linagliptin and positive values favour control.

The meta-analysis of two studies showed that linagliptin had no significant difference in GGT levels between linagliptin and control treatments as shown in Figure 5 [33,37]. The pooled MD was −0.44 U/L (95% CI: −6.80 to 5.93; *p* > 0.05), indicating no significant difference. Heterogeneity was low (*I*^2^ = 0%, *p* = 0.8828), suggesting highly consistent results. Individual studies also showed non-significant findings with confidence intervals crossing the null value. Overall, these results indicate that linagliptin does not significantly alter GGT levels compared with control interventions.

#### 3.5.2. Effect of Linagliptin on ALP

Only one study (Tian et al., 2023) reported ALP levels. At the end of treatment, the mean ALP level was 74.88 ± 21.02 U/L in the linagliptin group compared with 71.36 ± 21.48 U/L in the glimepiride group, and no statistically significant difference was observed in this study (*p* = 0.445) [37]. However, as ALP data were available from only one study, quantitative synthesis was not feasible, limiting the ability to determine the effect of linagliptin on ALP levels.

### 3.6. Subgroup Analysis of Primary Outcomes

#### 3.6.1. Subgroup Analysis of AST

**Figure 6:**
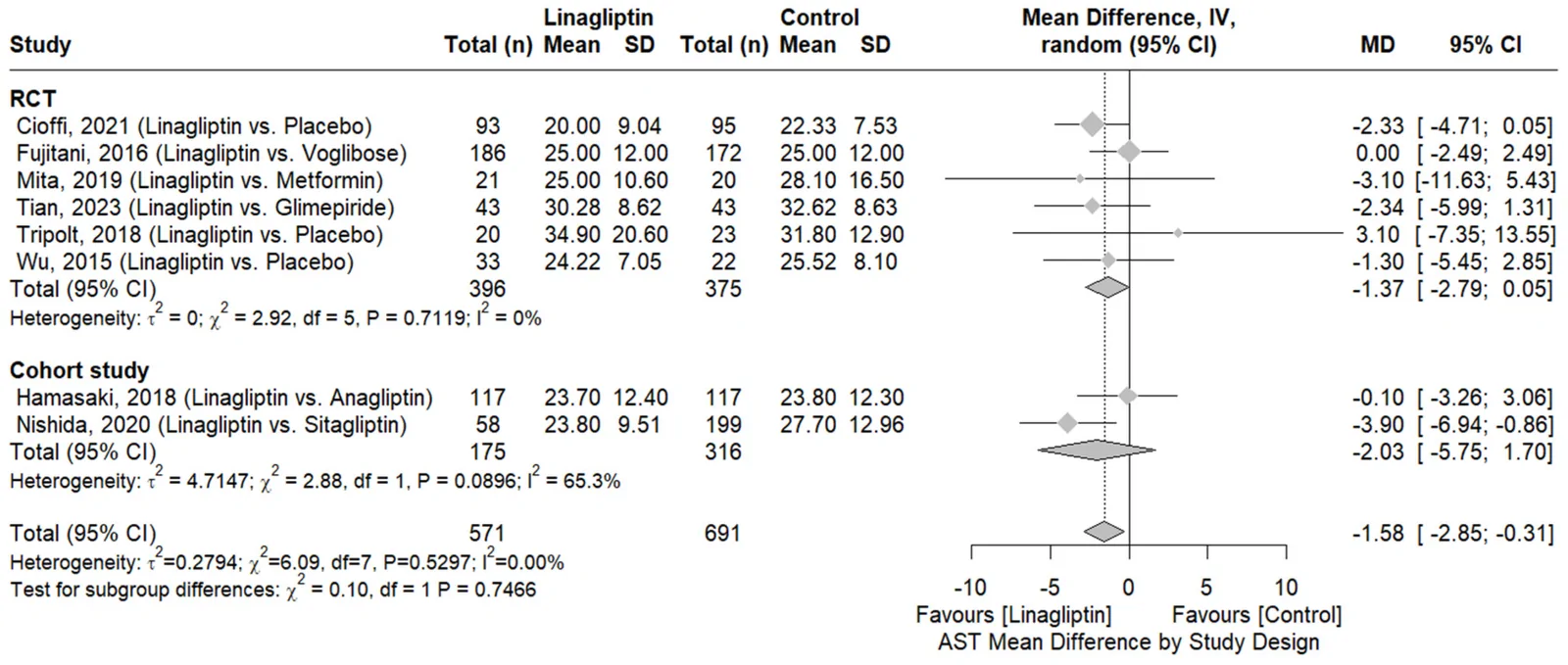
Subgroup analysis of the effect of linagliptin on aspartate aminotransferase (AST) levels according to study design (randomized controlled trials [32,33,35,37,38,39] vs. cohort studies [34,36]). Forest plot of mean differences (MDs) with 95% confidence intervals (CIs) based on a random-effects model. Squares indicate weighted study estimates, horizontal lines represent 95% CIs, and diamonds show pooled effects for subgroups and overall analysis. The vertical line at zero represents no effect; negative values favour linagliptin and positive values favour control.

When analyzed by study design (RCTs vs. cohort studies), reductions in AST levels with linagliptin were observed across both subgroups as shown in Figure 6. In RCTs, pooling six studies showed a non-significant reduction in AST levels (MD = −1.37 U/L, 95% CI: −2.79 to 0.05), with no heterogeneity (*I*^2^ = 0%, *p* = 0.7119) [32,33,35,37,38,39]. In cohort studies, pooling two studies demonstrated a greater but non-significant reduction in AST levels (MD = −2.03 U/L, 95% CI: −5.75 to 1.70), with substantial heterogeneity (*I*^2^ = 65.30%, *p* = 0.0896) [34,36]. The test for subgroup differences was not statistically significant (*p* = 0.7466), indicating that the effect of linagliptin on AST levels was consistent regardless of study design.

**Figure 7:**
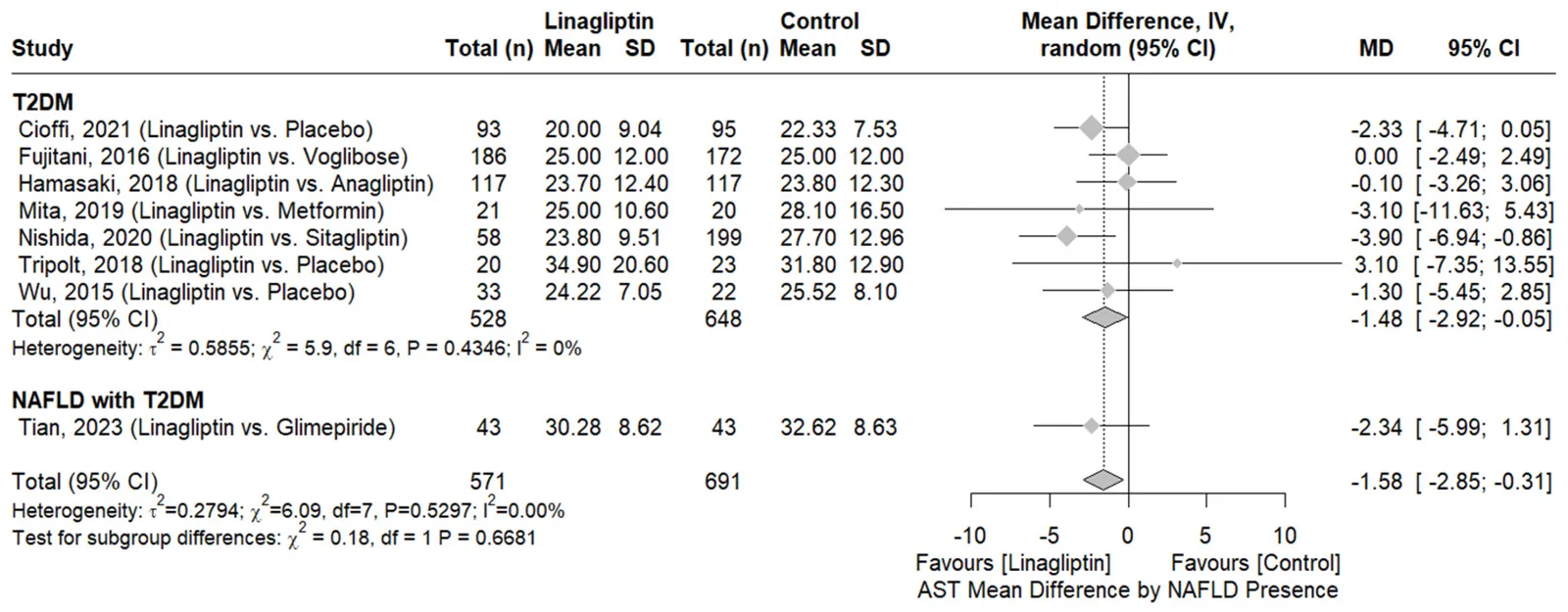
Subgroup analysis of the effect of linagliptin on aspartate aminotransferase (AST) levels according to disease status (T2DM [32,33,34,35,36,38,39] vs. NAFLD with T2DM [37]). Forest plot of MDs with 95% CIs based on a random-effects model. Squares represent weighted study estimates, horizontal lines denote 95% CIs, and diamonds indicate pooled effects for subgroups and the overall analysis. The vertical line at zero represents no effect; negative values favour linagliptin and positive values favour control.

Subgroup analysis based on disease status (T2DM vs. NAFLD with T2DM) showed that linagliptin was associated with a reduction in AST levels in both subgroups as shown in Figure 7. In patients with T2DM, the pooled analysis of seven studies demonstrated a significant decrease in AST levels (MD = −1.48 U/L, 95% CI: −2.92 to −0.05), with no evidence of heterogeneity (*I*^2^ = 0%, *p* = 0.4346) [32,33,34,35,36,38,39]. In contrast, in patients with NAFLD and T2DM (one study), linagliptin showed a non-significant reduction in AST levels compared with control (MD = −2.34 U/L, 95% CI: −5.99 to 1.31) [37]. No statistically significant difference was observed between the subgroups (*p* = 0.6681), suggesting that the effect of linagliptin was generally consistent across the subgroups. Overall, these findings suggest that the effect of linagliptin on AST levels is consistent across different patient populations.

**Figure 8:**
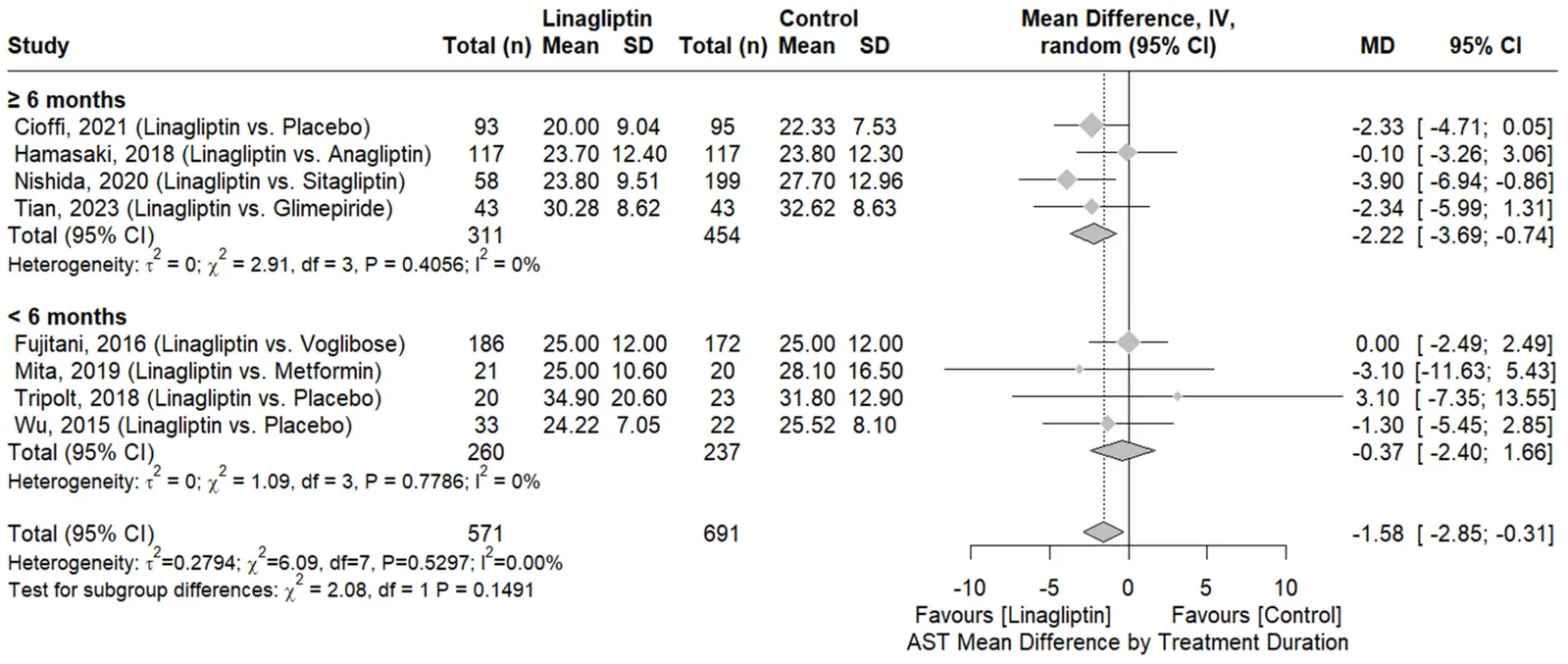
Subgroup analysis of the effect of linagliptin on aspartate aminotransferase (AST) levels according to treatment duration (≥6 months [32,34,36,37] vs. <6 months [33,35,38,39]). Forest plot showing mean differences (MDs) with 95% confidence intervals (CIs) derived from a random-effects model. Squares represent weighted study estimates, horizontal lines indicate 95% CIs, and diamonds show pooled effects for subgroups and overall analysis. The vertical line at zero represents no effect; negative values favour linagliptin and positive values favour control.

Subgroup analysis based on treatment duration (≥6 months vs. <6 months) demonstrated that the effect of linagliptin on AST differed by duration of treatment as shown in Figure 8. In studies with a treatment duration of ≥6 months, pooling four studies showed a significant reduction in AST levels (MD = −2.22 U/L, 95% CI: −3.69 to −0.74), with no evidence of heterogeneity (*I*^2^ = 0%, *p* = 0.4056) [32,34,36,37]. In contrast, in studies with a treatment duration of <6 months, pooling four studies demonstrated a non-significant reduction in AST levels (MD = −0.37 U/L, 95% CI: −2.40 to 1.66), also with no heterogeneity (*I*^2^ = 0%, *p* = 0.7786) [33,35,38,39]. No significant differences were observed across treatment-duration subgroups (*p* = 0.1491), indicating that the effect of linagliptin on AST levels was not substantially influenced by treatment duration.

#### 3.6.2. Subgroup Analysis of ALT

**Figure 9:**
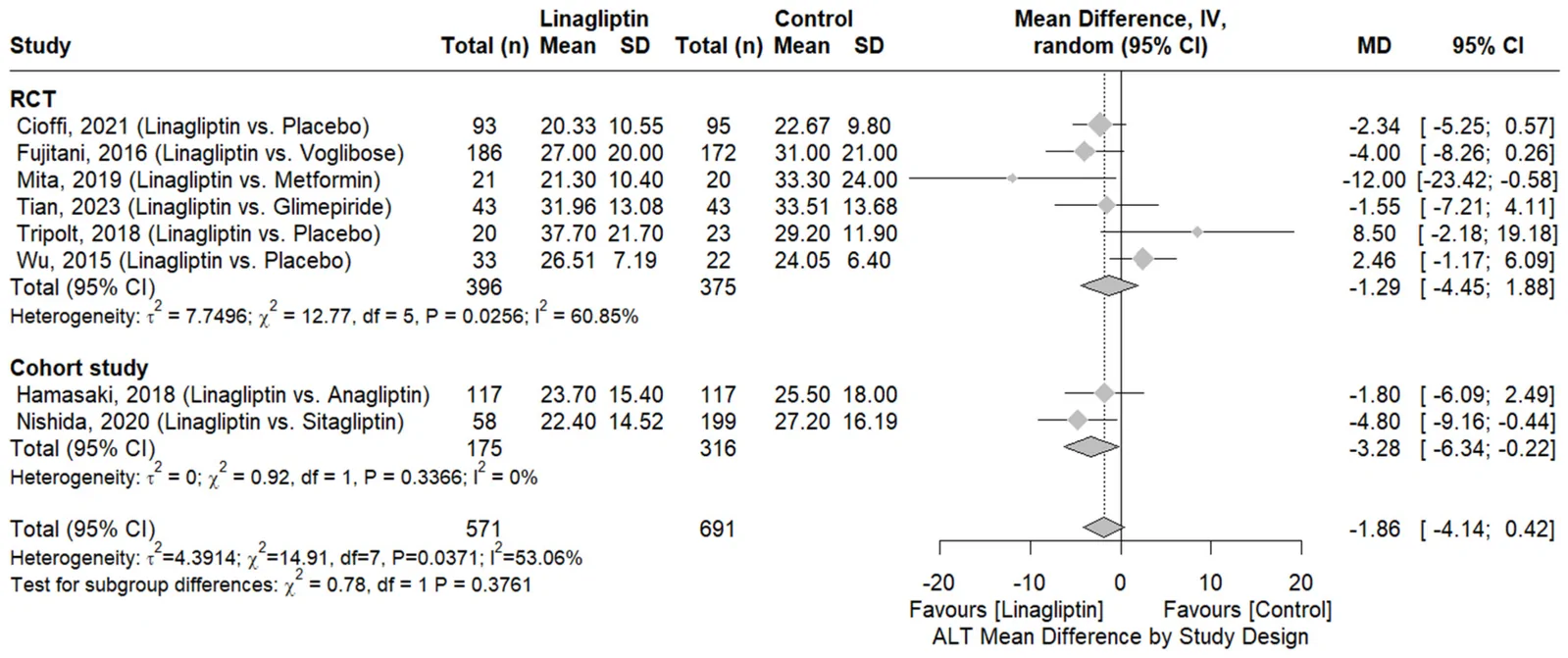
Subgroup analysis of the effect of linagliptin on alanine aminotransferase (ALT) levels stratified by study design (randomized controlled trials [32,33,35,37,38,39] vs. cohort studies [34,36]). Forest plot of MDs with 95% CIs based on a random-effects model. Squares represent weighted study estimates, horizontal lines denote 95% CIs, and diamonds indicate pooled effects for subgroups and overall analysis. The vertical line at zero represents no effect; negative values favour linagliptin and positive values favour control.

When stratified by study design, the impact of linagliptin on ALT levels showed differing patterns between RCTs and cohort studies as shown in Figure 9. Among RCTs, combining six studies revealed a modest but non-significant change in ALT levels (MD = −1.29 U/L, 95% CI: −4.45 to 1.88), with substantial variability across studies (*I*^2^ = 60.85%, *p* = 0.0256) [32,33,35,37,38,39]. In contrast, pooling two cohort studies demonstrated a significant decrease in ALT levels (MD = −3.28 U/L, 95% CI: −6.34 to −0.22), with no observed heterogeneity (*I*^2^ = 0%, *p* = 0.3366), indicating consistent findings within this subgroup [34,36]. No significant subgroup differences were observed (*p* = 0.3761), suggesting that study design did not affect the overall treatment effect.

**Figure 10:**
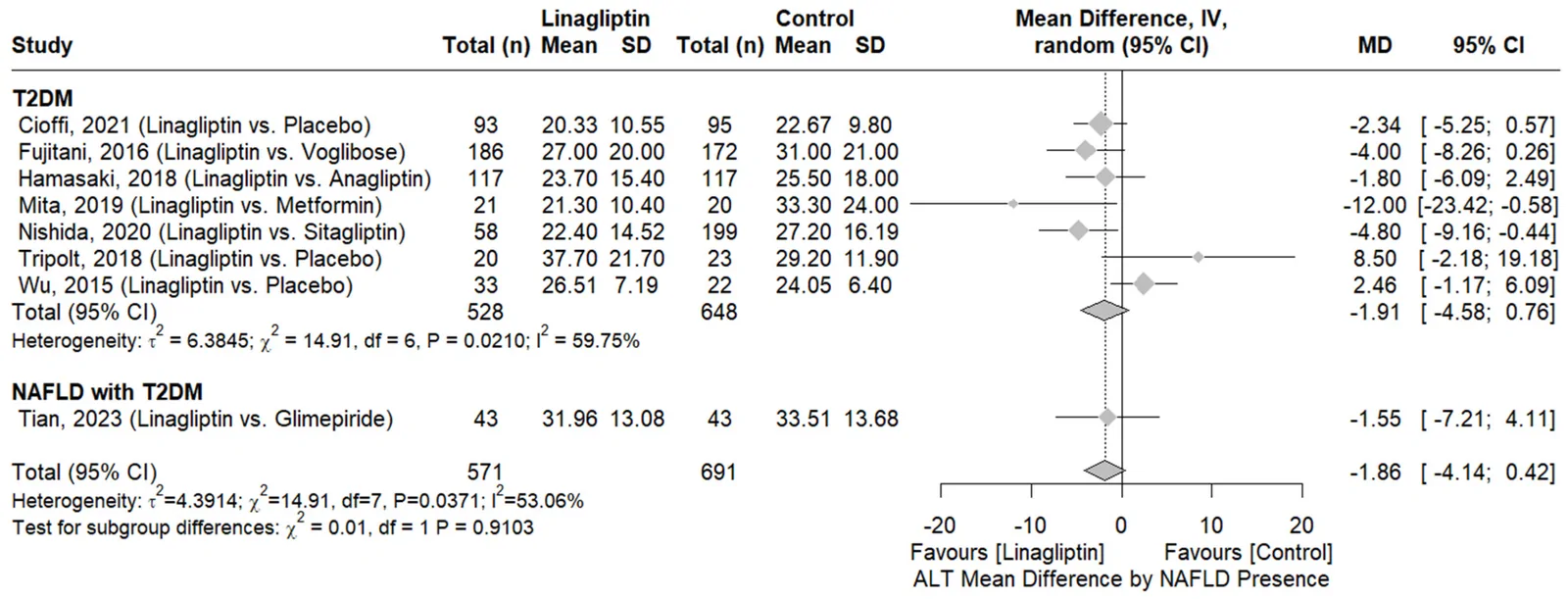
Subgroup analysis of the effect of linagliptin on alanine aminotransferase (ALT) levels according to disease status (T2DM [32,33,34,35,36,38,39] vs. NAFLD with T2DM [37]). Forest plot of mean differences (MDs) with 95% confidence intervals (CIs) based on a random-effects model. Squares represent weighted study estimates, horizontal lines denote 95% CIs, and diamonds indicate pooled effects for subgroups and overall analysis. The vertical line at zero represents no effect; negative values favour linagliptin and positive values favour control.

Subgroup analysis according to disease status (T2DM vs. NAFLD with T2DM) indicated that the effect of linagliptin on ALT levels was generally consistent across patient populations as shown in Figure 10. In individuals with T2DM, pooling seven studies showed a non-significant reduction in ALT levels (MD = −1.91 U/L, 95% CI: −4.58 to 0.76), with substantial heterogeneity (*I*^2^ = 59.75%, *p* = 0.0210) [32,33,34,35,36,38,39]. In contrast, data from a single study involving patients with NAFLD and T2DM demonstrated a non-significant decrease in ALT levels (MD = −1.55 U/L, 95% CI: −7.21 to 4.11) [37]. No significant subgroup differences were observed (*p* = 0.9103), suggesting that NAFLD status did not modify the effect on ALT levels.

**Figure 11:**
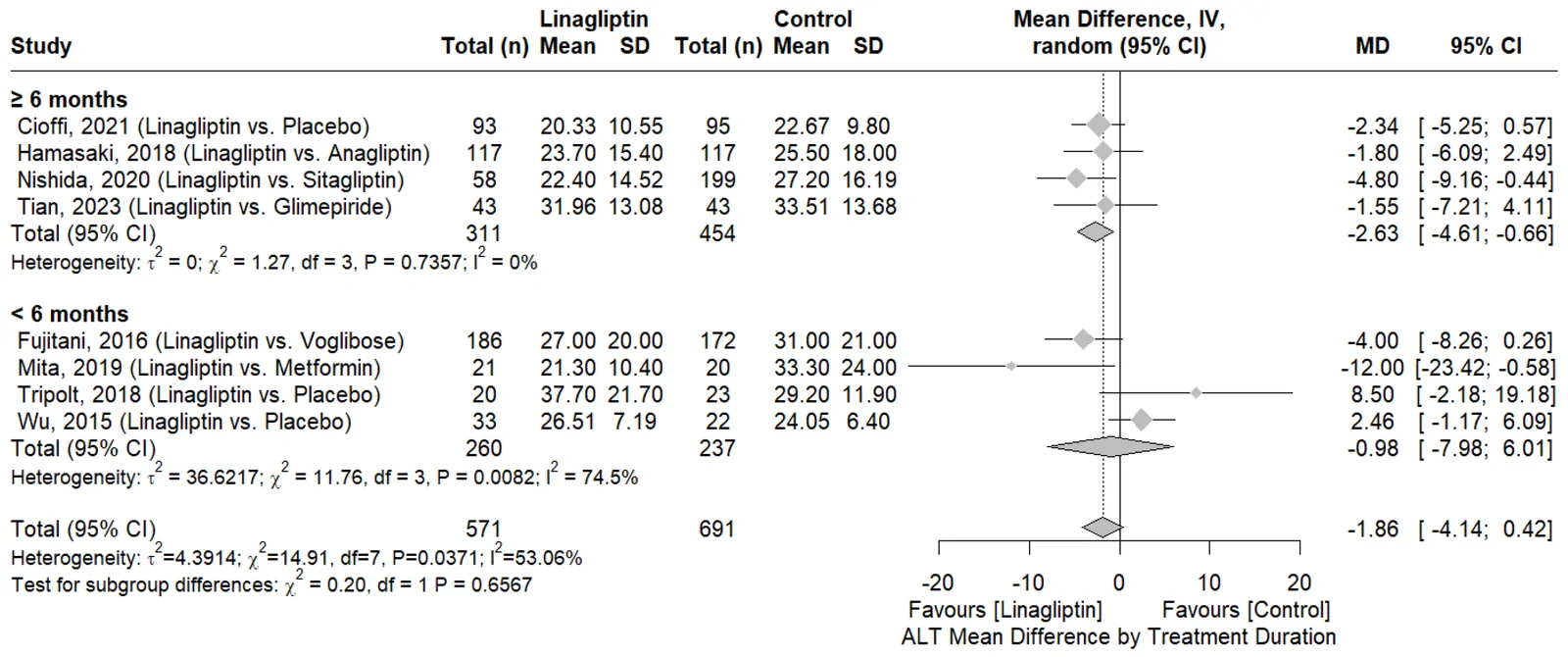
Subgroup analysis of the effect of linagliptin on alanine aminotransferase (ALT) levels according to treatment duration (≥6 months [32,34,36,37] vs. <6 months [33,35,38,39]). Forest plot of mean differences (MDs) with 95% confidence intervals (CIs) derived from a random-effects model. Squares indicate weighted study estimates, horizontal lines represent 95% CIs, and diamonds show pooled effects for subgroups and overall analysis. The vertical line at zero indicates no effect; negative values favour linagliptin and positive values favour control.

Subgroup analysis according to treatment duration (≥6 months vs. <6 months) indicated variation in the effect of linagliptin on ALT levels as shown in Figure 11. In studies with a treatment duration of ≥6 months, pooling four studies demonstrated a significant reduction in ALT levels (MD = −2.63 U/L, 95% CI: −4.61 to −0.66), with no heterogeneity observed (*I*^2^ = 0%, *p* = 0.7357) [32,34,36,37]. In contrast, studies with a treatment duration of <6 months (four studies) showed a non-significant change in ALT levels (MD = −0.98 U/L, 95% CI: −7.98 to 6.01), accompanied by substantial heterogeneity (*I*^2^ = 74.50%, *p* = 0.0082) [33,35,38,39]. No significant differences were found across treatment-duration subgroups (*p* = 0.6567), suggesting that the effect of linagliptin on ALT levels remained consistent regardless of treatment duration.

### 3.7. Sensitivity Analysis

**Figure 12:**
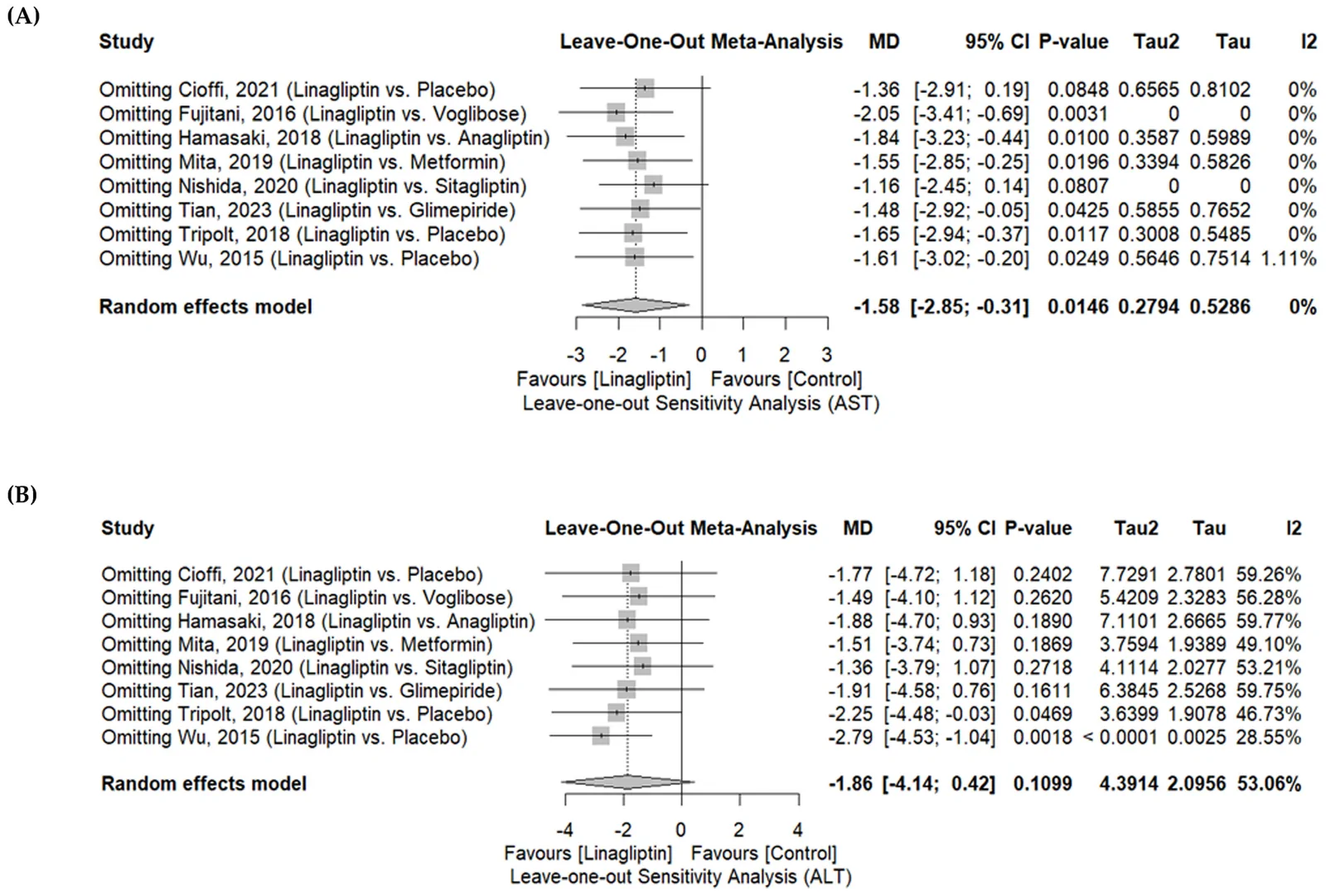
Leave-one-out sensitivity analysis of the effect of linagliptin on liver enzyme levels, including (**A**) aspartate aminotransferase (AST) and (**B**) alanine aminotransferase (ALT) [32,33,34,35,36,37,38,39]. Pooled mean differences (MDs) with 95% confidence intervals (CIs) were calculated using a leave-one-out random-effects model. The vertical line at zero represents no effect. Squares denote pooled estimates after exclusion of each study, and horizontal lines indicate 95% CIs. Negative values favour linagliptin and positive values favour control.

A leave-one-out sensitivity analysis was conducted to assess the influence of individual studies on the pooled estimates for both AST and ALT levels as shown in Figure 12. For AST (Figure 12A), the pooled mean differences (MDs) ranged from −1.16 to −2.05 U/L after sequential exclusion of each study. The results became non-significant when Cioffi et al., 2021 (MD = −1.36, 95% CI: −2.91 to 0.19; *p* = 0.0848), and Nishida et al., 2020 (MD = −1.16, 95% CI: −2.45 to 0.14; *p* = 0.0807), were omitted, as the confidence intervals crossed zero [32,36]. In contrast, omission of Fujitani et al., 2016; Hamasaki et al., 2018; Mita et al., 2019; Tian et al., 2023; Tripolt et al., 2018; or Wu et al., 2015, resulted in statistically significant reductions, with confidence intervals remaining below zero [33,34,35,37,38,39]. Overall, the direction of effect consistently favoured linagliptin, although statistical significance varied depending on the study removed.

For ALT (Figure 12B), the pooled MDs ranged from −1.36 to −2.79 U/L across the leave-one-out analyses. The results remained non-significant when Cioffi et al., 2021; Fujitani et al., 2016; Hamasaki et al., 2018; Mita et al., 2019; Nishida et al., 2020; and Tian et al., 2023, were excluded, as the corresponding confidence intervals included zero [32,33,34,35,36,37]. However, exclusion of Tripolt et al., 2018 (MD = −2.25, 95% CI: −4.48 to −0.03; *p* = 0.0469), and Wu et al., 2015 (MD = −2.79, 95% CI: −4.53 to −1.04; *p* = 0.0018), yielded statistically significant reductions in ALT levels [38,39]. Overall, these findings suggest that although the direction of effect was largely consistent, the statistical significance of the pooled estimates varied depending on the exclusion of individual studies, particularly for ALT.

### 3.8. Publication Bias

Due to the small number of eligible studies (*n* = 8), formal assessment of publication bias was not performed. Statistical methods commonly used to evaluate publication bias, such as funnel plot asymmetry and Egger’s regression test, are generally not recommended when fewer than 10 studies are available, as these methods have low power to distinguish chance from true asymmetry [40,41]. Visual inspection of funnel plots was therefore not performed, and any potential small-study effects or reporting bias could not be reliably evaluated. Consequently, the potential for publication bias cannot be excluded and should be considered when interpreting the overall results.

### 3.9. Results of GRADE Assessment of Evidence

The GRADE approach was used to assess the certainty of the available evidence as shown in Supplementary Table S11. For AST, evidence from eight studies (*n* = 1262) demonstrated a statistically significant reduction in AST levels with linagliptin compared with control (MD −1.58 U/L, 95% CI: −2.85 to −0.31), with no serious concerns across risk of bias, inconsistency, indirectness, or imprecision. As publication bias could not be assessed, the overall certainty of evidence was rated as moderate.

For ALT, pooled analysis of eight studies (*n* = 1262) showed a non-significant reduction (MD −1.86 U/L, 95% CI: −4.14 to 0.42). The certainty of the evidence was rated lower because of serious inconsistency, while other domains were not considered serious, resulting in an overall low certainty rating.

Two RCTs (*n* = 444) showed no significant difference in GGT levels between linagliptin and control (MD −0.44 U/L, 95% CI: −6.80 to 5.93). The certainty of evidence was very low due to serious imprecision, and publication bias was not assessed.

## 4. Discussion

Eight studies were included in this systematic review and meta-analysis (*n* = 1262 participants; 571 in the linagliptin group and 691 in the control group), comprising six randomized controlled trials and two cohort studies conducted across multiple regions, including Asia and Europe. The included populations predominantly consisted of patients with T2DM, with only one study involving individuals with concomitant NAFLD. The duration of treatment ranged from 3 to 24 months, and comparator interventions varied, including placebo, metformin, sulfonylureas, α-glucosidase inhibitors, and other dipeptidyl peptidase-4 (DPP-4) inhibitors. Overall, these findings suggest that the effect of linagliptin on liver enzyme levels was limited and varied across individual enzymes. The reduction in AST was modest and consistent, whereas no statistically significant changes were demonstrated for ALT or GGT, and the evidence for ALP was insufficient for a reliable assessment. Thus, the findings do not indicate a uniform effect of linagliptin across liver enzyme profiles.

The pooled analysis showed a statistically significant but small reduction in AST with linagliptin (MD −1.58 U/L, 95% CI −2.85 to −0.31; *I*^2^ = 0%). Although the confidence interval excluded the null value and the absence of heterogeneity indicated consistency across studies, the magnitude of the reduction was modest and should not be interpreted as evidence of clinically meaningful hepatic improvement. Previous evidence has shown that DPP-4 inhibitors can reduce AST levels in patients with T2DM and metabolic comorbidities, particularly those with NAFLD [42]. In a meta-analysis including patients with T2DM and NAFLD, DPP-4 inhibitor therapy was associated with significant reductions in AST, suggesting potential effects on liver enzyme levels in metabolically compromised populations. Similarly, broader analyses of glucose-lowering therapies have reported reductions in AST following treatment with DPP-4 inhibitors, although the magnitude of effect varies across populations and is influenced by baseline liver status and metabolic profiles [43]. However, contradictory evidence exists, as some systematic reviews have indicated that DPP-4 inhibitors may not significantly reduce AST compared with newer antidiabetic agents, such as GLP-1 receptor agonists or SGLT2 inhibitors, which appear to provide greater improvements in liver enzyme levels [44]. The clinical relevance of AST reduction may be greater in patients with elevated baseline AST or underlying MASLD/NAFLD, who have greater potential for biochemical improvement. However, baseline AST levels, the proportion of patients with elevated AST, and the stage and severity of MASLD were not consistently reported, and only one included study specifically enrolled patients with concurrent T2DM and NAFLD. Therefore, the present meta-analysis cannot determine whether the observed effect differs according to baseline AST or MASLD status, or whether the small reduction reflects a meaningful improvement in the underlying liver disease.

The pooled analysis for ALT demonstrated a non-significant reduction (MD −1.86 U/L, 95% CI: −4.14 to 0.42; *I*^2^ = 53.06%), indicating both statistical uncertainty and substantial heterogeneity across the included studies [32,33,34,35,36,37,38,39]. The confidence interval crossing the null value (zero effect) suggests that the observed reduction cannot be reliably distinguished from no effect, indicating that linagliptin does not consistently improve ALT levels in patients with T2DM. Despite the lack of statistical significance, the direction of effect generally favoured linagliptin, which may indicate a small underlying treatment effect that is insufficiently powered or diluted by variability across studies. This interpretation is supported by previous meta-analyses reporting significant reductions in ALT following treatment with DPP-4 inhibitors, particularly in patients with T2DM and NAFLD, where baseline liver enzyme abnormalities are more pronounced [42]. The substantial heterogeneity observed further suggests variability in treatment effects across populations and study conditions, which may influence the pooled estimate and contribute to the lack of statistical significance. In addition, broader evidence indicates that the effects of DPP-4 inhibitors on ALT are generally modest and inconsistent compared with those glucose-lowering therapies, particularly GLP-1 receptor agonists and SGLT2 inhibitors classes [43]. Indeed, comparative analyses have shown that these newer agents demonstrate greater and more consistent improvements in liver enzyme profiles than DPP-4 inhibitors [44]. Therefore, the non-significant pooled estimate and the substantial heterogeneity across studies indicate that the effect of linagliptin on ALT levels is not consistent. The wide confidence interval, which crosses the null value, further suggests uncertainty in the estimated treatment effect and indicates that the clinical relevance of this finding may be limited in the general T2DM population.

No significant difference in GGT levels was observed between the linagliptin and control groups in the pooled analysis (MD −0.44 U/L, 95% CI: −6.80 to 5.93; *I*^2^ = 0%), indicating a null effect with high consistency across the included studies [33,37]. Despite this consistency, the wide confidence interval crossing the null value reflects substantial imprecision and limited power, which reduces confidence in the effect estimate. The absence of a significant effect is consistent with previous systematic reviews suggesting that DPP-4 inhibitors generally exert minimal or inconsistent effects on GGT levels compared with other glucose-lowering agents [43,44]. In contrast, network meta-analyses have demonstrated that GLP-1 receptor agonists and SGLT2 inhibitors achieve greater reductions in GGT, indicating more pronounced effects on hepatic oxidative stress and cholestatic pathways [45]. These differences may reflect distinct pharmacological mechanisms, as DPP-4 inhibitors primarily improve glycemic control, whereas other drug classes additionally target hepatic fat accumulation, inflammation, and metabolic dysfunction [46]. Furthermore, broader systematic reviews of glucose-lowering therapies have shown that improvements in GGT are typically smaller and less consistent with DPP-4 inhibitors than with agents that directly reduce hepatic steatosis or body weight [43,45]. Taken together, the available evidence does not indicate a meaningful difference in GGT levels between linagliptin and control treatments.

For ALP, the evidence was limited to a single study demonstrating no statistically significant difference in enzyme levels between linagliptin and control groups (*p* = 0.445), precluding meta-analysis and limiting the strength of inference [37]. This paucity of data is consistent with the existing literature, in which ALP is infrequently reported as an outcome in trials evaluating DPP-4 inhibitors; therefore, evidence from the single-study does not allow a reliable conclusion regarding the effect of linagliptin on ALP levels [43]. Moreover, ALP is a non-specific biomarker influenced by multiple physiological processes, including hepatobiliary function, bone turnover, and systemic metabolic conditions, which may reduce its sensitivity in detecting treatment-related changes in T2DM populations [46]. In addition, the relatively short duration of most included studies may not be sufficient to detect meaningful changes in cholestatic enzymes, which may respond more slowly to metabolic interventions [42]. Consequently, the current evidence remains insufficient to draw definitive conclusions regarding the effect of linagliptin on ALP, and further well-designed studies with larger sample sizes and longer follow-up are warranted to clarify its potential impact.

Subgroup analyses of AST and ALT provide insight into the consistency and potential clinical relevance of the observed effects of linagliptin across different study conditions. Notably, the reduction in AST appeared more consistent across subgroups, with minimal heterogeneity and comparable effect estimates regardless of study design, patient population, and treatment duration, suggesting a relatively stable hepatic response to linagliptin. In contrast, ALT results demonstrated greater variability, particularly in RCTs and shorter-duration studies, which may reflect the known biological and analytical fluctuations of ALT levels as well as differences in patient characteristics, baseline liver status, and metabolic profiles. This discrepancy between AST and ALT responses has also been observed in the previous literature, where ALT tends to be more sensitive to metabolic and inflammatory changes in non-alcoholic fatty liver disease and type 2 diabetes, leading to greater heterogeneity in clinical studies [43]. The finding that longer treatment duration (≥6 months) was associated with more pronounced reductions in both AST and ALT further supports a time-dependent hepatic benefit, which is consistent with clinical observations that improvement in liver enzymes often requires sustained metabolic control [47,48]. Overall, while the subgroup analyses did not reveal statistically significant effect modification, the patterns observed highlight the potential influence of study design, patient population, and treatment duration on ALT variability. The longer treatment duration was also associated with greater reductions in AST and ALT. These findings should nevertheless be interpreted with caution, given the small number of available studies and differences in study characteristics.

The leave-one-out sensitivity analysis showed that the pooled results remained largely unchanged after excluding each study in turn, as the direction of effect consistently favoured linagliptin across all iterations, and exclusion of individual studies resulted in only minor changes in the pooled estimates, indicating that the overall effect was not substantially influenced by any individual study. However, fluctuations in statistical significance, particularly for ALT, indicate that the precision of the pooled estimates is sensitive to individual study characteristics, and this greater variability in ALT is consistent with previous evidence showing that liver enzymes may exhibit substantial biological and clinical variability in patients with metabolic disorders, contributing to heterogeneity across studies [49]. In contrast, AST findings appear relatively more stable and support the robustness of the observed trend, and prior studies have reported that DPP-4 inhibitor therapy produces modest but consistent improvements in liver enzymes over time, although the magnitude of change may vary depending on study design and population [47]. Taken together, these findings suggest that the direction of changes in liver enzyme levels generally favoured linagliptin; however, the statistical significance, particularly for ALT, should be interpreted with caution because of underlying variability and study-level influences.

The included studies were generally considered to be of high methodological quality as most RCTs demonstrated a low risk of bias across key domains, and the cohort studies were rated as good quality according to established assessment tools, supporting the internal validity of the findings. However, some concerns were noted in a few studies, particularly related to missing outcome data and selective reporting, which may have introduced minor uncertainty into the pooled estimates. Because only a small number of studies were available, publication bias could not be formally assessed. Consequently, the possibility of small-study effects or selective publication cannot be excluded. Furthermore, the GRADE assessment indicated moderate certainty for AST outcomes, suggesting that the observed effect is likely to be reliable, whereas the certainty for ALT was downgraded to low due to inconsistency, reflecting the observed heterogeneity across studies. The evidence for GGT was rated as very low, primarily owing to imprecision and limited data availability. Taken together, these findings indicate that while the evidence supporting the effect of linagliptin on liver enzymes is generally of acceptable quality, the overall confidence in the results varies by outcome, and cautious interpretation is warranted, particularly for outcomes with lower certainty.

Linagliptin exerts its beneficial effects on liver enzymes through multiple complementary mechanisms that extend beyond glycemic control, primarily via inhibition of DPP-4 activity and subsequent enhancement of incretin signaling. By preventing the degradation of glucagon-like peptide-1 (GLP-1), linagliptin increases insulin secretion and suppresses glucagon release in a glucose-dependent manner, thereby improving systemic and hepatic insulin sensitivity. Improved insulin sensitivity reduces hepatic de novo lipogenesis and promotes lipid oxidation, which ultimately decreases hepatic fat accumulation, a key driver of liver injury in type 2 diabetes mellitus. In addition, DPP-4 inhibition has been associated with anti-inflammatory and antifibrotic effects through downregulation of pro-inflammatory cytokines and modulation of hepatic immune responses. Experimental and clinical evidence further suggests that linagliptin may improve hepatic lipid metabolism and reduce oxidative stress, thereby attenuating hepatic steatosis and contributing to improved liver enzyme profiles [13]. Moreover, clinical studies have demonstrated that DPP-4 inhibitor therapy is associated with modest but consistent reductions in AST and ALT levels over time, supporting the clinical relevance of these mechanistic pathways [41].

Several interconnected physiological and molecular mechanisms may explain the observed reduction in liver enzymes following linagliptin therapy. First, as a DPP-4 inhibitor, linagliptin prolongs the half-life of GLP-1 [50]. Elevated levels of active GLP-1 enhance insulin sensitivity, optimize lipid metabolism, and directly reduce hepatic lipotoxicity by downregulating de novo lipogenesis and promoting fatty acid β-oxidation in hepatocytes [17,50]. Consequently, the reduction in intracellular lipid accumulation mitigates hepatocellular stress and cellular ballooning. Second, beyond its effects on glycemic and lipid regulation, linagliptin exhibits distinct anti-inflammatory and antioxidative properties. Chronic hyperglycemia induces activation of the nuclear factor-kappa B (NF-κB) signaling pathway in the liver, leading to increased production of pro-inflammatory cytokines, including tumor necrosis factor-alpha (TNF-α) and interleukin-6 (IL-6) [17]. Linagliptin has been shown to suppress macrophage infiltration into hepatic tissue and downregulate these inflammatory pathways [17,51]. By preserving hepatocyte membrane integrity and reducing cell necrosis, the leakage of intracellular AST and ALT into the systemic circulation is minimized. Furthermore, direct inhibition of DPP-4 activity in hepatic stellate cells may attenuate early fibrogenic signaling, which may contribute to the observed changes in liver enzyme levels [51].

Several limitations of this study should be acknowledged when interpreting the findings. First, the relatively limited number of included studies may constrain the broader applicability of the findings, particularly for subgroup and sensitivity analyses. Second, although most studies were of moderate to high methodological quality, some concerns regarding missing data and selective reporting may introduce residual bias. Third, heterogeneity was observed, particularly in the ALT analyses, which may be attributable to differences in study design, patient populations, baseline hepatic status, and comparator interventions. Fourth, follow-up periods differed among the included studies, potentially influencing the observed treatment effects, as improvements in liver enzymes may require sustained therapy. Fifth, the inclusion of randomized controlled trials and observational studies may contribute to variability in effect estimates despite subgroup analyses. Sixth, publication bias could not be formally assessed due to the limited number of studies, and therefore the possibility of unpublished or small-study effects cannot be excluded. Seventh, only one included study specifically enrolled patients with concurrent T2DM and NAFLD, whereas the remaining studies predominantly included broader T2DM populations without detailed characterization of baseline liver disease. Baseline liver enzyme abnormalities and the stage and severity of MASLD/NAFLD were also not consistently reported. Consequently, the findings cannot be reliably extrapolated to patients with T2DM and established liver injury or used to determine whether the effects of linagliptin vary according to MASLD stage or severity. Liver enzyme outcomes were used as nonspecific surrogate markers and do not directly reflect changes in hepatic steatosis, inflammation, fibrosis, or structural liver damage. Histological findings, imaging-based assessments, fibrosis measures, and clinically relevant hepatic outcomes were not consistently available across studies. Therefore, the observed reduction in AST cannot be interpreted as direct evidence of improvement in the underlying liver disease or a hepatoprotective effect of linagliptin. The evidence for ALP was also limited, as only one included study reported this outcome, precluding quantitative synthesis and limiting the ability to draw conclusions regarding the effect of linagliptin on ALP levels. Finally, differences in reporting formats, such as mean ± standard deviation versus median with interquartile range, may introduce minor estimation bias during data conversion. In addition, the available studies mainly assessed liver enzyme levels during active treatment, and no data were available on whether the reduction in AST persisted after linagliptin discontinuation. Future studies should therefore include patients with well-characterized stages of MASLD and longer follow-up periods, including assessments after treatment discontinuation, to determine whether the observed changes in liver enzyme levels are sustained and whether the treatment effects differ according to disease stage. Taken together, these limitations suggest that while the findings provide valuable insights into the potential effects of linagliptin on liver enzyme levels, cautious interpretation is warranted.

Overall, this systematic review and meta-analysis suggest that linagliptin is associated with modest improvements in liver enzyme levels, particularly for AST, with a consistent direction of effect across included studies. Although reductions in ALT were observed, the findings were less robust and demonstrated greater variability. Subgroup and sensitivity analyses further supported the overall stability of the results, while highlighting the influence of study characteristics on statistical significance, especially for ALT. Despite the generally acceptable methodological quality of the included studies, limitations such as small sample size, heterogeneity, and the use of surrogate liver biomarkers underscore the need for cautious interpretation. Future large-scale, well-designed studies with longer follow-up and comprehensive hepatic outcomes are warranted to confirm these findings and to better define the clinical relevance of linagliptin in the management of liver dysfunction in patients with T2DM.

## 5. Conclusions

In conclusion, linagliptin was associated with a modest reduction in AST, but the clinical significance of this finding remains uncertain. No statistically significant effects were observed for ALT or GGT, and the evidence for ALP was insufficient. Because only one study included patients with concurrent T2DM and NAFLD and no histological or imaging outcomes were available, these findings cannot confirm a hepatoprotective effect or be reliably extrapolated to patients with established liver injury. Further studies involving well-characterized MASLD populations, longer follow-up periods, and comprehensive hepatic outcomes are needed.

## Figures and Tables

**Table 1:** Study eligibility criteria defined according to the PICOS framework.

Parameter	Description
Population (P)	Adult patients (≥18 years old) clinically diagnosed with type 2 diabetes mellitus (T2DM) without restriction on the presence or absence of baseline hepatic conditions, such as metabolic dysfunction-associated steatotic liver disease (MASLD) or non-alcoholic fatty liver disease (NAFLD).
Intervention (I)	Monotherapy with linagliptin at any therapeutic dose and for any follow-up duration.
Comparison (C)	Control groups receiving a placebo, standard clinical care, or other active antidiabetic therapies (including metformin, insulin, or alternative DPP-4 inhibitors).
Outcomes (O)	Primary outcome: alanine aminotransferase (ALT) and aspartate aminotransferase (AST). Secondary outcomes: alkaline phosphatase (ALP) and gamma-glutamyl transferase (GGT).
Study Design (S)	Randomized controlled trials (RCTs), non-randomized controlled trials, and observational cohort studies of both prospective and retrospective designs.

**Table 2:** Characteristics of the included studies evaluating the effects of linagliptin on liver enzymes in patients with type 2 diabetes mellitus (T2DM).

Study (Author, Year)	Country	Study Design	Patient Population	Treatment Duration (Months)	Patient, N (Linagliptin/Control)	Control	Outcomes	Value
Cioffi, 2021 [32]	Italy	RCT	T2DM	12	93/95	Placebo	AST, ALT	Median (IQR)
Fujitani, 2016 [33]	Japan	RCT	T2DM	3	186/172	Voglibose	AST, ALT, GGT	Mean ± SD
Hamasaki, 2018 [34]	Japan	Cohort study	T2DM	24	117/117	Anagliptin	AST, ALT	Mean ± SD
Mita, 2019 [35]	Japan	RCT	T2DM	6	21/20	Metformin	AST, ALT	Mean ± SD
Nishida, 2020 [36]	Japan	Cohort study	T2DM	12	58/199	Sitagliptin	AST, ALT	Mean ± SD
Tian, 2023 [37]	China	RCT	NAFLD with T2DM	6	43/43	Glimepiride	AST, ALT, ALP, GGT	Mean ± SD
Tripolt, 2018 [38]	Austria	RCT	T2DM	3	20/23	Placebo	AST, ALT	Mean ± SD
Wu, 2015 [39]	China	RCT	T2DM	6	33/22	Placebo	AST, ALT	Mean ± SD

ALP, alkaline phosphatase; ALT, alanine aminotransferase; AST, aspartate aminotransferase; GGT, gamma-glutamyl transferase; IQR, interquartile range; NAFLD, non-alcoholic fatty liver disease; RCT, randomized controlled trial; SD, standard deviation; T2DM, type 2 diabetes mellitus.

**Table 3:** Quality assessment of the included cohort studies based on the Newcastle–Ottawa Scale (NOS) and Agency for Healthcare Research and Quality (AHRQ) criteria [34,36].

Author (Year)	Selection (Max: 4 ★)	Comparability (Max: 2 ★)	Outcome (Max: 3 ★)	Total Stars	AHRQ Rating
Hamasaki, 2018 [34]	★★★★	★	★★	7/9	Good quality
Nishida, 2020 [36]	★★★★	★★	★★★	9/9	Good quality

Stars (★) indicate the score assigned according to the Newcastle–Ottawa Scale, with higher scores indicating better methodological quality.

## Data Availability

All data generated or analyzed during this study are included in this published article and its Supplementary Materials.

## Supplementary Materials

The following supporting information can be downloaded at https://www.mdpi.com/article/10.3390/life16081343/s1: Table S1: Search strategy for PubMed; Table S2: Search strategy for Scopus; Table S3: Search strategy for the Cochrane Library; Table S4: Search strategy for Web of Science; Table S5: Search strategy for Embase (via Ovid); Table S6: Inter-rater agreementfor title and abstract screening; Table S7: Inter-rater agreement for full-text screening; Table S8: Extracted outcome data from the included studies; Table S9: Conversion of median and interquartile range to estimated mean and standard deviation for Cioffi et al., 2021 [32]; Table S10: Newcastle–Ottawa Scale (NOS) assessment of included studies across three domains; Table S11: Summary of findings for the effect of linagliptin compared with control treatments on liver enzymes (AST, ALT, and GGT) in patients with type 2 diabetes mellitus (T2DM).

## Abbreviations

The following abbreviations are used in this manuscript:

AHRQ	Agency for Healthcare Research and Quality
AI	Artificial Intelligence
ALP	Alkaline Phosphatase
ALT	Alanine Aminotransferase
AST	Aspartate Aminotransferase
CI	Confidence Interval
D1–D5	RoB 2 Domains 1 to 5
GGT	Gamma-Glutamyl Transferase
GRADE	Grading of Recommendations Assessment, Development and Evaluation
*I*^2^	I-squared Heterogeneity Statistic
IQR	Interquartile range
MASLD	Metabolic Dysfunction-Associated Steatotic Liver Disease
MD	Mean Difference
n	Sample Size/Number of Studies
NAFLD	Non-Alcoholic Fatty Liver Disease
NF-κB	Nuclear factor-kappa B
NOS	Newcastle–Ottawa Scale
*p*	*p*-value/Probability Value
PRISMA	Preferred Reporting Items for Systematic Reviews and Meta-Analyses
RCT	Randomized Controlled Trial
RoB 2	Cochrane Risk of Bias 2 Tool
SD	Standard deviation
T2DM	Type 2 Diabetes Mellitus
α	Alpha Level/Significance Level
*κ*	Cohen’s Kappa Coefficient

## References

[1] American Diabetes Association (2026). Standards of care in diabetes—2026. Diabetes Care.

[2] DeFronzo R.A., Eldor R., Abdul-Ghani M. (2015). Type 2 diabetes mellitus. Nat. Rev. Dis. Primers.

[3] Targher G., Byrne C.D., Tilg H. (2021). Non-alcoholic fatty liver disease: A multisystem disease requiring a multidisciplinary and holistic approach. Lancet Diabetes Endocrinol..

[4] Paschos P., Paletas K. (2009). Non alcoholic fatty liver disease and metabolic syndrome. Hippokratia.

[5] European Association for the Study of the Liver (2024). EASL-EASD-EASO Clinical Practice Guidelines on the management of metabolic dysfunction-associated steatotic liver disease (MASLD). J. Hepatol..

[6] Younossi Z.M., Golabi P., de Avila L. (2023). The global epidemiology of nonalcoholic fatty liver disease (NAFLD) and nonalcoholic steatohepatitis (NASH). Hepatology.

[7] Lee C., Lui D., Lam K. (2022). Non-alcoholic fatty liver disease and type 2 diabetes: An update. J. Diabetes Investig..

[8] American Diabetes Association (2025). Standards of care in diabetes—2025. Diabetes Care.

[9] Giannini E.G., Testa R., Savarino V. (2005). Liver enzyme alteration: A guide for clinicians. Can. Med. Assoc. J..

[10] Pratt D.S., Kaplan M.M. (2000). Evaluation of abnormal liver-enzyme results in asymptomatic patients. N. Engl. J. Med..

[11] Gallwitz B. (2019). Clinical Use of DPP-4 Inhibitors. Front. Endocrinol..

[12] Deacon C.F. (2019). Physiology and Pharmacology of DPP-4 in Glucose Homeostasis and the Treatment of Type 2 Diabetes. Front. Endocrinol..

[13] Aloke C., Adelusi O.A., Onisuru O.O., Iwuchukwu E.A., Achilonu I. (2025). Dipeptidyl peptidase 4 inhibitors: Novel therapeutic agents in the management of type II diabetes mellitus. Pharmacoepidemiol. Drug Saf..

[14] Gallwitz B. (2013). Emerging DPP-4 inhibitors: Focus on linagliptin for type 2 diabetes. Diabetes Metab. Syndr. Obes..

[15] Michurina S.V., Ishenko I.J., Klimontov V.V., Archipov S.A., Myakina N.E., Cherepanova M.A., Zavjalov E.L., Koncevaya G.V., Konenkov V.I. (2016). Linagliptin alleviates fatty liver disease in diabetic db/db mice. World J. Diabetes.

[16] Okuyama T., Shirakawa J., Tajima K., Ino Y., Vethe H., Togashi Y., Kyohara M., Inoue R., Miyashita D., Li J. (2020). Linagliptin ameliorates hepatic steatosis via non-canonical mechanisms in mice treated with a dual inhibitor of insulin receptor and IGF-1 receptor. Int. J. Mol. Sci..

[17] Kern M., Klöting N., Niessen H.G. (2012). Linagliptin improves insulin sensitivity and hepatic steatosis in diet-induced obesity. PLoS ONE.

[18] Klein T., Fujii M., Sandel J. (2014). Linagliptin alleviates hepatic steatosis and inflammation in a mouse model of non-alcoholic steatohepatitis. Med. Mol. Morphol..

[19] Page M.J., McKenzie J.E., Bossuyt P.M., Boutron I., Hoffmann T.C., Mulrow C.D., Shamseer L., Tetzlaff J.M., Akl E.A., Brennan S.E. (2021). The PRISMA 2020 statement: An updated guideline for reporting systematic reviews. BMJ.

[20] Ouzzani M., Hammady H., Fedorowicz Z., Elmagarmid A. (2016). Rayyan—A web and mobile app for systematic reviews. Syst. Rev..

[21] McHugh M.L. (2012). Interrater reliability: The kappa statistic. Biochem. Med..

[22] Sterne J.A.C., Savović J., Page M.J., Elbers A.G., Blencowe N.S., Boutron I., Cates C.J., Cheng J.H., Corbett M.S., Eldridge S.M. (2019). RoB 2: A revised tool for assessing risk of bias in randomised trials. BMJ.

[23] Gualdi-Russo E., Zaccagni L. (2026). The Newcastle–Ottawa scale for assessing the quality of studies in systematic reviews. Publications.

[24] Viswanathan M., Ansari M.H., Berkman N.D., Chang S., Hartling L., McPheeters M.L., Santaguida P.L. (2012). Assessing the Risk of Bias of Individual Studies in Systematic Reviews of Health Care Interventions.

[25] Guyatt G.H., Oxman A.D., Vist G.E., Kunz R., Brozek J., Alonso-Coello P., Montori V., Akl E.A., Djulbegovic B., Falck-Ytter Y. (2011). GRADE guidelines: 4. Rating the quality of evidence—Study limitations (risk of bias). J. Clin. Epidemiol..

[26] Luo D., Wan X., Liu J., Tong T. (2018). Optimally estimating the sample mean from the sample size, median, mid-range, and/or mid-quartile range. Stat. Methods Med. Res..

[27] DerSimonian R., Laird N. (1986). Meta-analysis in clinical trials. Control. Clin. Trials.

[28] Higgins J.P.T., Thompson S.G., Deeks J.J., Altman D.G. (2003). Measuring inconsistency in meta-analyses. BMJ.

[29] Meng Z., Wang J., Lin L., Wu C. (2024). Sensitivity analysis with iterative outlier detection for systematic reviews and meta-analyses. Stat. Med..

[30] Begg C.B., Mazumdar M. (1994). Operating characteristics of a rank correlation test for publication bias. Biometrics.

[31] Egger M., Smith G.D., Schneider M., Minder C. (1997). Bias in meta-analysis detected by a simple, graphical test. BMJ.

[32] Cioffi G., Giorda C.B., Lucci D., Misuraca G., Ognibeni F., Gulizia M.M., Tarantini L., Di Lenarda A., Maggioni A.P. (2021). Effects of linagliptin on left ventricular DYsfunction in patients with type 2 DiAbetes and concentric left ventricular geometry: Results of the DYDA 2 trial. Eur. J. Prev. Cardiol..

[33] Fujitani Y., Fujimoto S., Takahashi K., Kawamori R., Watada H. (2016). Effects of linagliptin monotherapy compared with voglibose on postprandial blood glucose responses in Japanese patients with type 2 diabetes: Linagliptin Study of Effects on Postprandial blood glucose (L-STEP). Diabetes Res. Clin. Pract..

[34] Hamasaki H., Hamasaki Y. (2018). Efficacy of anagliptin as compared to linagliptin on metabolic parameters over 2 years of drug consumption: A retrospective cohort study. World J. Diabetes.

[35] Mita T., Hiyoshi T., Yoshii H., Zhao C., Okada Y., Osonoi T., Karasawa S., Saito M., Kanazawa A., Watada H. (2019). The effect of linagliptin versus metformin treatment related quality of life in patients with type 2 diabetes mellitus. Diabetes Ther..

[36] Nishida Y., Takahashi Y., Tezuka K., Akimoto H., Nakayama T., Asai S. (2020). Comparative effect of dipeptidyl-peptidase 4 inhibitors on laboratory parameters in patients with diabetes mellitus. BMC Pharmacol. Toxicol..

[37] Tian F., Guo Y., Zhou L., Wang X. (2023). Comparison of glimepiride and linagliptin in the treatment of non-alcoholic hepatic disease with type 2 diabetes mellitus. Arch. Med. Sci..

[38] Tripolt N.J., Aberer F., Riedl R., Sourij H. (2018). Effects of linagliptin on endothelial function and postprandial lipids in coronary artery disease patients with early diabetes: A randomized, placebo-controlled, double-blind trial. Cardiovasc. Diabetol..

[39] Wu W., Li Y., Chen X., Zhang L. (2015). Effect of linagliptin on glycemic control in Chinese patients with newly-diagnosed, drug naïve type 2 diabetes mellitus: A randomized controlled trial. Med. Sci. Monit..

[40] Cumpston M., Li T., Page M.J., Chandler J., Welch V.A., Higgins J.P.T., Thomas J. (2019). Updated guidance for trusted systematic reviews: A new edition of the Cochrane Handbook for Systematic Reviews of Interventions. Cochrane Database Syst. Rev..

[41] Sterne J.A., Sutton A.J., Ioannidis J.P., Terrin N., Jones D.R., Lau J., Carpenter J., Rücker G., Harbord R.M., Schmid C.H. (2011). Recommendations for examining and interpreting funnel plot asymmetry in meta-analyses of randomised controlled trials. BMJ.

[42] Ma G., Zhang S., Yu B. (2025). Impact of dipeptidyl peptidase-4 inhibitors on aminotransferases levels in patients with type 2 diabetes mellitus with nonalcoholic fatty liver disease: A meta-analysis of randomized controlled trial. Curr. Ther. Res. Clin. Exp..

[43] Fu Z.D., Cai X.L., Yang W.J., Zhao M.M., Li R., Li Y.F. (2021). Novel glucose-lowering drugs for non-alcoholic fatty liver disease. World J. Diabetes.

[44] Zafar Y., Rashid A.M., Siddiqi A.K., Ellahi A., Ahmed A., Hussain H.U., Ahmed F., Menezes R.G., Siddiqi T.J., Maniya M.T. (2022). Effect of novel glucose lowering agents on non-alcoholic fatty liver disease: A systematic review and meta-analysis. Clin. Res. Hepatol. Gastroenterol..

[45] Kongmalai T., Srinonprasert V., Anothaisintawee T., Kongmalai P., McKay G., Attia J., Thakkinstian A. (2023). New anti-diabetic agents for the treatment of non-alcoholic fatty liver disease: A systematic review and network meta-analysis of randomized controlled trials. Front. Endocrinol..

[46] Sharma A., Virmani T., Sharma A., Chhabra V., Kumar G., Pathak K., Alhalmi A. (2022). Potential effect of DPP-4 inhibitors towards hepatic diseases and associated glucose intolerance. Diabetes Metab. Syndr. Obes..

[47] Kanazawa I., Tanaka K., Sugimoto T. (2014). DPP-4 inhibitors improve liver dysfunction in type 2 diabetes mellitus. Med. Sci. Monit..

[48] Haxhi J., Vitale M., Mattia L., Giuliani C., Sacchetti M., Orlando G., Iacobini C., Menini S., Zanuso S., Nicolucci A. (2024). Effect of sustained decreases in sedentary time and increases in physical activity on liver enzymes and indices in type 2 diabetes. Front. Endocrinol..

[49] Bang K., Jun J.E., Jeong I.K., Ahn K.J., Chung H.Y., Hwang Y.C. (2021). Increased visit-to-visit liver enzyme variability is associated with incident diabetes: A community-based 12-year prospective cohort study. Diabetes Metab. J..

[50] Drucker D.J. (2018). Mechanisms of action and therapeutic application of glucagon-like peptide-1. Cell Metab..

[51] Kaji K., Yoshiji H., Ikenaka Y., Noguchi R., Aihara Y., Douhara A., Kawaratani H., Fukui H. (2014). Dipeptidyl peptidase-4 inhibitor attenuates hepatic fibrosis via suppression of activated hepatic stellate cell in rats. J. Gastroenterol..

