# Supplementary material for: Effects of Linagliptin on Liver Enzymes in Patients with Type 2 Diabetes Mellitus: A Systematic Review and Meta-Analysis
Source: Life (Basel). 2026 Aug 16;16(8):1343. doi: 10.3390/life16081343 (PMC13514491; doi:10.3390/life16081343)
Supplement: Supplementary file 1 [file life-16-01343-s001.zip › life-4423961-supplementary.pdf]

# Effects of Linagliptin on Liver Enzymes in Patients with Type 2 Diabetes Mellitus: A Systematic Review and Meta-analysis

**Moragot Chatatikun [1,2,3], Ratana Netphakdee [2], Aman Tedasen [1,2,3], Jason C. Huang [4], Wiyada Kwanhian Klangbud [5], Jongkonnee Thanasai [6] and Atthaphong Phongphithakchai [7,*]**

1 Department of Medical Technology, School of Allied Health Sciences, Walailak University, Nakhon Si Thammarat 80160, Thailand; moragot.ch@wu.ac.th; aman.te@wu.ac.th
2 Biomedical Sciences and Health Innovations (International Program), School of Allied Health Sciences, Walailak University, Nakhon Si Thammarat 80160, Thailand; ratana.ne@mail.wu.ac.th
3 Research Excellence Center for Innovation and Health Products (RECIHP), Walailak University, Nakhon Si Thammarat 80160, Thailand
4 Department of Biotechnology and Laboratory Science in Medicine, National Yang Ming Chiao Tung University, Taipei 112304, Taiwan; jasonhuang@nycu.edu.tw
5 Medical Technology Program, Faculty of Science, Nakhon Phanom University, Nakhon Phanom 48000, Thailand; wiydakhanhian@gmail.com
6 Faculty of Medicine, Mahasarakham University, Mahasarakham 44000, Thailand; jongkonnee@msu.ac.th
7 Nephrology Unit, Division of Internal Medicine, Faculty of Medicine, Prince of Songkla University, Songkhla 90110, Thailand
* Correspondence: atthaphong.p@psu.ac.th (A.P.)

**Table S1.** Search strategy for PubMed.

| Search Number | Query | Search Details | Results |
|---|---|---|---|
| 1 | "Diabetes Mellitus, Type 2"[MeSH] | "diabetes mellitus, type 2"[MeSH Terms] | 199,212 |
| 2 | type 2 diabetes[Title/Abstract] | "type 2 diabetes"[Title/Abstract] | 211,299 |
| 3 | type 2 diabetes mellitus[Title/Abstract] | "type 2 diabetes mellitus"[Title/Abstract] | 81,626 |
| 4 | type II diabetes[Title/Abstract] | "type ii diabetes"[Title/Abstract] | 11,257 |
| 5 | type II diabetes mellitus[Title/Abstract] | "type ii diabetes mellitus"[Title/Abstract] | 3,993 |
| 6 | T2DM[Title/Abstract] | "T2DM"[Title/Abstract] | 47,015 |
| 7 | non-insulin dependent diabetes[Title/Abstract] | "non insulin dependent diabetes"[Title/Abstract] | 8,801 |
| 8 | NIDDM[Title/Abstract] | "NIDDM"[Title/Abstract] | 7,012 |
| 9 | diabet*[Title/Abstract] | "diabet*"[Title/Abstract] | 930,007 |
| 10 | (((((((("Diabetes Mellitus, Type 2"[MeSH]) OR (type 2 diabetes[Title/Abstract])) OR (type 2 diabetes mellitus[Title/Abstract])) OR (type II diabetes[Title/Abstract])) OR (type II diabetes mellitus[Title/Abstract])) OR (T2DM[Title/Abstract])) OR (non-insulin dependent diabetes[Title/Abstract])) OR (NIDDM[Title/Abstract])) OR (diabet*[Title/Abstract]) | "diabetes mellitus, type 2"[MeSH Terms] OR "type 2 diabetes"[Title/Abstract] OR "type 2 diabetes mellitus"[Title/Abstract] OR "type ii diabetes"[Title/Abstract] OR "type ii diabetes mellitus"[Title/Abstract] OR "T2DM"[Title/Abstract] OR "non insulin dependent diabetes"[Title/Abstract] OR "NIDDM"[Title/Abstract] OR "diabet*"[Title/Abstract] | 943,919 |
| 11 | Linagliptin[MeSH] | "linagliptin"[MeSH Terms] | 703 |
| 12 | linagliptin[Title/Abstract] | "linagliptin"[Title/Abstract] | 1,220 |
| 13 | BI 1356[Title/Abstract] | "bi 1356"[Title/Abstract] | 23 |
| 14 | DPP-4 inhibitor*[Title/Abstract] | "dpp 4 inhibitor*"[Title/Abstract] | 3,901 |
| 15 | dipeptidyl peptidase-4 inhibitor*[Title/Abstract] | "dipeptidyl peptidase 4 inhibitor*"[Title/Abstract] | 4,137 |
| 16 | ((((Linagliptin[MeSH]) OR (linagliptin[Title/Abstract])) OR (BI 1356[Title/Abstract])) OR (DPP-4 | "linagliptin"[MeSH Terms] OR "linagliptin"[Title/Abstract] OR "bi 1356"[Title/Abstract] OR "dpp 4 | 7,395 |

| | | | |
|---|---|---|---|
| | inhibitor*[Title/Abstract])) OR (dipeptidyl peptidase-4 inhibitor*[Title/Abstract]) | inhibitor*"[Title/Abstract] OR "dipeptidyl peptidase 4 inhibitor*"[Title/Abstract] | |
| 17 | ((((((((("Diabetes Mellitus, Type 2"[MeSH]) OR (type 2 diabetes[Title/Abstract])) OR (type 2 diabetes mellitus[Title/Abstract])) OR (type II diabetes[Title/Abstract])) OR (type II diabetes mellitus[Title/Abstract])) OR (T2DM[Title/Abstract])) OR (non-insulin dependent diabetes[Title/Abstract])) OR (NIDDM[Title/Abstract])) OR (diabet*[Title/Abstract])) AND (((((Linagliptin[MeSH]) OR (linagliptin[Title/Abstract])) OR (BI 1356[Title/Abstract])) OR (DPP-4 inhibitor*[Title/Abstract])) OR (dipeptidyl peptidase-4 inhibitor*[Title/Abstract])) | ("diabetes mellitus, type 2"[MeSH Terms] OR "type 2 diabetes"[Title/Abstract] OR "type 2 diabetes mellitus"[Title/Abstract] OR "type ii diabetes"[Title/Abstract] OR "type ii diabetes mellitus"[Title/Abstract] OR "T2DM"[Title/Abstract] OR "non insulin dependent diabetes"[Title/Abstract] OR "NIDDM"[Title/Abstract] OR "diabet*"[Title/Abstract]) AND ("linagliptin"[MeSH Terms] OR "linagliptin"[Title/Abstract] OR "bi 1356"[Title/Abstract] OR "dpp 4 inhibitor*"[Title/Abstract] OR "dipeptidyl peptidase 4 inhibitor*"[Title/Abstract]) | 6,457 |

**Table S2.** Search strategy for Scopus.

| Search Number | Search Details | Results |
|---|---|---|
| 1 | TITLE-ABS-KEY ( type 2 diabetes ) | 387,259 |
| 2 | TITLE-ABS-KEY ( type 2 diabetes mellitus ) | 342,506 |
| 3 | TITLE-ABS-KEY ( type II diabetes ) | 42,418 |
| 4 | TITLE-ABS-KEY ( type II diabetes mellitus ) | 35,709 |
| 5 | TITLE-ABS-KEY ( T2DM ) | 54,744 |
| 6 | TITLE-ABS-KEY ( non insulin dependent diabetes ) | 347,277 |
| 7 | TITLE-ABS-KEY ( NIDDM ) | 8,783 |
| 8 | TITLE-ABS-KEY ( diabet* ) | 1,523,700 |
| 9 | ( ( TITLE-ABS-KEY ( type 2 diabetes ) ) OR ( TITLE-ABS-KEY ( type 2 diabetes mellitus ) ) OR ( TITLE-ABS-KEY ( type II diabetes ) ) OR ( TITLE-ABS-KEY ( T2DM ) ) OR ( TITLE-ABS-KEY ( non insulin dependent diabetes ) ) OR ( TITLE-ABS-KEY ( NIDDM ) ) OR ( TITLE-ABS-KEY ( diabet* ) ) ) OR ( TITLE-ABS-KEY ( type II diabetes mellitus ) ) | 1,524,284 |
| 10 | TITLE-ABS-KEY ( linagliptin ) | 4,070 |
| 11 | TITLE-ABS-KEY ( BI 1356 ) | 91 |
| 12 | TITLE-ABS-KEY ( DPP-4 inhibitor* ) | 5,404 |
| 13 | TITLE-ABS-KEY ( dipeptidyl peptidase-4 inhibitor* ) | 8,210 |
| 14 | ( TITLE-ABS-KEY ( linagliptin ) ) OR ( TITLE-ABS-KEY ( BI 1356 ) ) OR ( TITLE-ABS-KEY ( DPP-4 inhibitor* ) ) OR ( TITLE-ABS-KEY ( dipeptidyl peptidase-4 inhibitor* ) ) | 12,327 |
| 15 | ( ( TITLE-ABS-KEY ( type 2 diabetes ) ) OR ( TITLE-ABS-KEY ( type 2 diabetes mellitus ) ) OR ( TITLE-ABS-KEY ( type II diabetes ) ) OR ( TITLE-ABS-KEY ( type II diabetes mellitus ) ) OR ( TITLE-ABS-KEY ( T2DM ) ) OR ( TITLE-ABS-KEY ( non insulin dependent diabetes ) ) OR ( TITLE-ABS-KEY ( NIDDM ) ) OR ( TITLE-ABS-KEY ( diabet* ) ) ) AND ( ( TITLE-ABS-KEY ( linagliptin ) ) OR ( TITLE-ABS-KEY ( BI 1356 ) ) OR ( TITLE-ABS-KEY ( DPP-4 inhibitor* ) ) OR ( TITLE-ABS-KEY ( dipeptidyl peptidase-4 inhibitor* ) ) ) | 10,286 |

**Table S3**. Search strategy for the Cochrane Library.

| Search Number | Search Details | Results |
|---|---|---|
| #1 | ("type 2 diabetes"):ti,ab,kw OR ("type 2 diabetes mellitus"):ti,ab,kw OR ("type II diabetes"):ti,ab,kw OR ("type II diabetes mellitus"):ti,ab,kw OR (T2DM):ti,ab,kw OR ("non insulin dependent diabetes"):ti,ab,kw OR ("NIDDM"):ti,ab,kw OR ("diabet"):ti,ab,kw | 56803 |
| #2 | ("linagliptin"):ti,ab,kw OR ("BI 1356"):ti,ab,kw OR ("DPP-4 inhibitor*"):ti,ab,kw OR ("dipeptidyl peptidase-4 inhibitor*"):ti,ab,kw | 2394 |
| #3 | #1 AND #2 | 1974 |

**Table S4**. Search strategy for Web of Science.

| Query | Results |
|---|---|
| (TI=("type 2 diabetes" OR "type 2 diabetes mellitus" OR "type II diabetes" OR "type II diabetes mellitus" OR T2DM OR "non insulin dependent diabetes" OR NIDDM OR diabet*) OR AB=("type 2 diabetes" OR "type 2 diabetes mellitus" OR "type II diabetes" OR "type II diabetes mellitus" OR T2DM OR "non insulin dependent diabetes" OR NIDDM OR diabet*)) AND (TI=(linagliptin OR "BI 1356" OR "DPP-4 inhibitor*" OR "dipeptidyl peptidase-4 inhibitor*") OR AB=(linagliptin OR "BI 1356" OR "DPP-4 inhibitor*" OR "dipeptidyl peptidase-4 inhibitor*")) | 6,514 |

**Table S5**. Search strategy for Embase (via Ovid).

| Search Number | Query | Results |
|---|---|---|
| 1 | type 2 diabetes.ab,kf,ti. | 342,138 |
| 2 | type 2 diabetes mellitus.ab,kf,ti. | 127,259 |
| 3 | type II diabetes.ab,kf,ti. | 20,106 |
| 4 | type II diabetes mellitus.ab,ti. | 7,387 |
| 5 | T2DM.ab,kf,ti. | 79,808 |
| 6 | non insulin dependent diabetes.ab,kf,ti. | 10,809 |
| 7 | NIDDM.ab,kf,ti. | 8,479 |
| 8 | "diabet*".ab,kf,ti. | 1,468,874 |
| 9 | 1 or 2 or 3 or 4 or 5 or 6 or 7 or 8 | 1,472,663 |
| 10 | linagliptin.ab,kf,ti. | 2,469 |
| 11 | BI 1356.ab,kf,ti. | 78 |
| 12 | "DPP-4 inhibitor*".ab,kf,ti. | 7,165 |
| 13 | "dipeptidyl peptidase-4 inhibitor*".ab,kf,ti. | 5,914 |
| 14 | 10 or 11 or 12 or 13 | 12,631 |
| 15 | 9 and 14 | 10,896 |

**Table S6**. Inter-rater agreement for title and abstract screening.

| **Cohen's Kappa (κ = 0.94)** | **Rater 2 (M.C.): Exclude** | **Rater 2 (M.C.): Include** | **Total Rater 1 (R.N.)** |
|---|---|---|---|
| Rater 1 (R.N.): Exclude | 11,650 | 20 | 11,670 |
| Rater 1 (R.N.): Include | 13 | 250 | 263 |
| Total Rater 2 (M.C.) | 11,663 | 270 | 11,936 |

**Table S7**. Inter-rater agreement for full-text screening.

| Cohen's Kappa (κ = 0.83) | Rater 2 (M.C.): Exclude | Rater 2 (M.C.): Include | Total Rater 1 (R.N.) |
|---|---|---|---|
| Rater 1 (R.N.): Exclude | 246 | 1 | 247 |
| Rater 1 (R.N.): Include | 2 | 8 | 10 |
| Total Rater 2 (M.C.) | 248 | 9 | 257 |

**Table S8.** Extracted outcome data from the included studies.

| Study (Author, Year) | Number of Patients (Linagliptin/Control) | AST | ALT | ALP | GGT | Control | AST | ALT | ALP | GGT | Value |
|---|---|---|---|---|---|---|---|---|---|---|---|
| Cioffi, 2021 | 93/95 | 20 [16–28] | 19 [14–28] | - | - | Placebo | 21 [18–28] | 21 [17–30] | - | - | Median (IQR) |
| Fujitani, 2016 | 186/172 | 25 ± 12 | 27 ± 20 | - | 45 ± 43 | Voglibose | 25 ± 12 | 31 ± 21 | - | 46 ± 51 | Mean ± SD |
| Hamasaki, 2018 | 117/117 | 23.7 (12.4) | 23.7 (15.4) | - | - | Anagliptin | 23.8 (12.3) | 25.5 (18) | - | - | Mean ± SD |
| Mita, 2019 | 21/20 | 25.0 ± 10.6 | 21.3 ± 10.4 | - | - | Metformin | 28.1 ± 16.5 | 33.3 ± 24.0 | - | - | Mean ± SD |
| Nishida, 2020 | 58/199 | 23.8 ± 9.51 | 22.4 ± 14.52 | - | - | Sitagliptin | 27.7 (12.96) | 27.2 (16.19) | - | - | Mean ± SD |
| Tian, 2023 | 43/43 | 30.28 ± 8.62 | 31.96 ± 13.08 | 74.88 ± 21.02 | 27.63 ± 15.81 | Glimepiride | 32.62 ± 8.63 | 33.51 ± 13.68 | 71.36 ± 21.48 | 27.66 ± 23.52 | Mean ± SD |
| Tripolt, 2018 | 20/23 | 34.9 ± 20.6 | 37.7 ± 21.7 | - | - | Placebo | 31.8 ± 12.9 | 29.2 ± 11.9 | - | - | Mean ± SD |
| Wu, 2015 | 33/22 | 24.22 ± 7.05 | 26.51 ± 7.19 | - | - | Placebo | 25.52 ± 8.10 | 24.05 ± 6.40 | - | - | Mean ± SD |

**Table S9.** Conversion of median and interquartile range to estimated mean and standard deviation for Cioffi et al., 2021.

| Study | Outcome | Group | *n* | Original Median | Original IQR | Estimated Mean | Estimated SD |
|---|---|---|---|---|---|---|---|
| Cioffi et al., 2021 | AST | Linagliptin | 93 | 20 | [16–28] | 20 | 9.04 |
| Cioffi et al., 2021 | AST | Control | 95 | 21 | [18–28] | 22.33 | 7.53 |
| Cioffi et al., 2021 | ALT | Linagliptin | 93 | 19 | [14–28] | 20.33 | 10.55 |
| Cioffi et al., 2021 | ALT | Control | 95 | 21 | [17–30] | 22.67 | 9.8 |

Values reported as median (interquartile range) in Cioffi et al. (2021) were converted to approximate mean and standard deviation for quantitative synthesis. The conversion was performed using the method described by Luo et al. [26], which provides an approach for estimating the sample mean from reported median and distributional information. The original median and interquartile range and the corresponding estimated mean and standard deviation are presented to allow verification of the data transformation used in the meta-analysis.

**Table S10**. Newcastle–Ottawa Scale (NOS) assessment of included studies across three domains.

| Author (Year) | Selection (Max: 4★) | | | | Comparability (Max: 2★) | Outcome (Max: 3★) | | | Total Stars | AHRQ Quality Rating |
|---|---|---|---|---|---|---|---|---|---|---|
| | Exposed Cohort/ Adequate Case Definition | Nonexposed Cohort/ Representativeness of The Cases | Ascertainment of Exposure/ Selection of Controls | Outcome of Interest/ Definition of controls | | Assessment of Outcome/Exposure | Length of Follow Up/Same Method for Cases and Controls | Adequacy of Follow Up/Non Response Rate | | |
| Hamasaki et al., 2018 [3] | ★ | ★ | ★ | ★ | ★ | ★ | ★ | – | 7/9 | Good quality |
| Nishida et al., 2020 [5] | ★ | ★ | ★ | ★ | ★★ | ★ | ★ | ★ | 9/9 | Good quality |

NOS evaluates methodological quality across three domains: selection (maximum 4 stars), comparability (maximum 2 stars), and outcome (maximum 3 stars), yielding a maximum total of nine stars. Studies with higher star ratings indicate better methodological quality. AHRQ quality ratings were assigned as good, fair, or poor according to predefined criteria based on domain-specific star allocation. A hyphen (–) indicates that the criterion was not applicable or not reported.

**Table S11**. Summary of findings for the effect of linagliptin compared with control treatments on liver enzymes (AST, ALT, and GGT) in patients with type 2 diabetes mellitus (T2DM).

| Certainty Assessment | | | | | | | Participants (Intervention/ Control) | Effect | | Certainty |
|---|---|---|---|---|---|---|---|---|---|---|
| No. of Studies | Study Design | Risk of Bias | Inconsistency | Indirectness | Imprecision | Other Considerations | | Relative Effect (95% CI) | Absolute Effect | |
| **AST** | | | | | | | | | | |
| 8 studies ($n$ = 1,262) | RCT + Cohort | Not serious[a] | Not serious[b] | Not serious[c] | Not serious[d] | Publication bias not assessable[e] | 571/691 | MD −1.58 U/L (−2.85 to −0.31) | 1.58 U/L lower | ⊕⊕⊕◯ Moderate |
| **ALT** | | | | | | | | | | |
| 8 studies ($n$ = 1,262) | RCT + Cohort | Not serious[a] | Serious[b] | Not serious[c] | Not serious[d] | Publication bias not assessable[e] | 571/691 | MD −1.86 U/L (−4.14 to 0.42) | No significant difference | ⊕⊕◯◯ Low |
| **GGT** | | | | | | | | | | |
| 2 studies ($n$ = 444) | RCT | Not serious[a] | Not serious[b] | Not serious[c] | Very serious[d] | Publication bias not assessable[e] | 229/215 | MD −0.44 U/L (−6.80 to 5.93) | No significant difference | ⊕◯◯◯ Very Low |

**Explanations**

[a] **Risk of bias:** Most included studies were judged to have low or moderate risk of bias, with no major methodological limitations affecting the overall estimate.

[b] **Inconsistency:** For AST and GGT, heterogeneity was low and results were consistent across studies; for ALT, substantial heterogeneity was observed ($I^2$ = 53.06%), leading to downgrading.

[c] **Indirectness:** The populations, interventions, comparators, and outcomes were directly relevant to the review question, with no serious concerns regarding applicability.

[d] **Imprecision:** For AST and ALT, confidence intervals were relatively narrow with adequate sample sizes; for GGT, wide confidence intervals crossing the null value and small sample size resulted in very serious imprecision.

[e] **Publication bias:** Publication bias could not be formally assessed due to the limited number of included studies (< 10).
